# Assessing “Friendly Fire”: The development & validation of the Cultural Betrayal Multidimensional Inventory for Black American Young Adults (CBMI-BAYA)

**DOI:** 10.1371/journal.pmen.0000537

**Published:** 2026-04-08

**Authors:** Jennifer M. Gómez, Lars U. Johnson

**Affiliations:** 1 School of Social Work, Boston University, Boston, Massachusetts, United States of America; 2 African American & Black Diaspora Studies, Boston University, Boston, Massachusetts, United States of America; 3 Center for Innovation in Social Work & Health, Boston University, Boston, Massachusetts, United States of America; 4 Department of Management, The University of Texas at Arlington, Arlington, Texas, United States of America; Public Library of Science, UNITED KINGDOM OF GREAT BRITAIN AND NORTHERN IRELAND

## Abstract

Racism impacts Black American young adults and further impacts those who experience violence. Cultural betrayal trauma theory proposes (intra)cultural trust, such as solidarity in the Black community, is a protective factor against racism. Because it violates (intra)cultural trust, within-group violence is a cultural betrayal trauma that negatively impacts mental health and cultural outcomes, such as internalized prejudice. An ongoing limitation of the evidence base is that there are no validated measures of aspects of the theory. The purpose of the current multi-study report is to develop and validate the Cultural Betrayal Multidimensional Inventory for Black American Young Adults, with questionnaires for (intra)cultural trust, cultural betrayal, violence & discrimination, (intra)cultural pressure, (intra)cultural support, and posttraumatic growth. Following creating questionnaires based on the literature and incorporating content expert feedback, we administered the Cultural Betrayal Multidimensional Inventory for Black American Young Adults and convergent/divergent validity measures to Black university students (Study 1; *N* = 342) and Black community young adults (Study 2; *N* = 619). Confirmatory factor analyses, correlational analyses, content expert feedback, and theoretical contribution review by the authors resulted in a scientifically valid, culturally congruent multidimensional inventory for assessing cultural betrayal trauma theory specifically—and violence in the context of racism generally—with Black American young adults. The Cultural Betrayal Multidimensional Inventory for Black American Young Adults can engender research on violence, discrimination, and in-group dynamics with Black American young adults, which has implications for basic research that can inform culturally competent violence prevention and intervention programs.

## Introduction

Though the impact of violence victimization is well-documented (e.g., [1,2]), the context of anti-Black racism and other forms inequality (e.g., misogynoir, or sexism against Black women and girls; [3,4]) additionally impacts outcomes [5–13]. To aid in calls to meaningfully incorporate such context into violence research (e.g., [14–17]), Gómez created cultural betrayal trauma theory (CBTT; e.g., [18,19]), as a framework for empirically testing how the “friendly fire” of cultural betrayal—that is, violence that is perpetrated by other Black people—impacts mental, physical, behavioral, and cultural health and wellness. Though the research is promising (*see* [20], *for a systematic review*), there remains methodological issues with this work. Specifically, adapted measures that have not been validated with Black populations have been used, with some constructs of CBTT excluded entirely from the research due to lack of validated measures. Therefore, the purpose of the current multi-study report is to develop and validate the Cultural Betrayal Multidimensional Inventory for Black American Young Adults (CBMI-BAYA) with two samples of Black American young adults.

### Violence victimization against the Black community

Violence victimization includes physical, sexual, and psychological abuse, which are linked to varied outcomes, including poor mental health (e.g., *see* [1], for a review). The transition to adulthood, approximately ages 18–30 years, confers additional risk for victimization (e.g., [2]) and mental health struggles (e.g., [21,22]). Moreover, within the context of structural and cultural anti-Black racism [23–28], Black American young adults also witness and experience racial discrimination and police violence (e.g., [23]), along with additional forms of victimization, such as sex trafficking, at alarmingly high rates [29]. Therefore, a theoretical framework that includes the context of racism is needed to better understand violence sequelae at a basic science level, which ultimately can inform culturally congruent interventions and healing strategies for Black young adults who experience violence.

### Cultural betrayal trauma theory

Cultural betrayal trauma theory (CBTT; e.g., [18,19]) builds upon Black feminist scholarship (e.g., [4,30]), ethnic minority trauma psychology (e.g., [8]), and betrayal trauma theory (e.g., [31,32]) to empirically examine the impact of violence within the context of inequality. Specifically, CBTT details how the context of societal trauma, such as anti-Black racism, engenders (intra)cultural trust for some members of the Black community. Such (intra)cultural trust in the Black community is broadly defined as Black solidarity, including love, loyalty, connection, and responsibility with other Black people (e.g., [11]). Consequently, within-group violence in which the perpetrator(s) and victim(s) are Black implicitly includes a cultural betrayal, as such violence is a violation of (intra)cultural trust. Moreover, cultural betrayal traumas—within-group violence in the Black community—are proposed to be linked with typically-studied abuse outcomes, such as mental health, as well as understudied cultural outcomes, such as internalized prejudice (e.g., [19,20,33]).

Finally, an additional component of CBTT is (intra)cultural pressure. Similar to racial loyalty (e.g., [5]), (intra)cultural pressure is a negative transformation of (intra)cultural trust in which the perceived needs of the perpetrator(s) and/or the Black community as a whole are privileged over the needs of the person(s) victimized (e.g., [19]). In practice, this includes promoting silence when cultural betrayal traumas occur in order to protect Black people from further prejudice and discrimination within systems, including K-12 education [34,35], policing [36], mental health care [24], policy [37], the criminal justice system [38], and media [39,40].

The empirical research with diverse marginalized young adults (e.g., [41,42]) lends support for CBTT. Specifically, this work has shown that cultural betrayal trauma predicts poor mental health, such as PTSD and depression, and cultural outcomes, such as internalized prejudice and changes to racial identity, for marginalized survivors [43,44]. These findings hold when controlling for between-group violence (e.g., White perpetrator; [33,45–49]) and the perpetrator being someone trusted and/or depended upon [47,48,50].

Though this research is promising (e.g., [20]), it is limited in multiple ways [19]. First, the research does not focus exclusively on Black American young adult populations. Second, important facets of CBTT, such as (intra)cultural trust and (intra)cultural pressure, are assessed using adapted measures that were initially validated without CBTT in mind (e.g., [46]). Third, the aforementioned studies have not included certain types of violence that may impact Black American young adults (e.g., [51]), such as police violence, racial discrimination, and sex trafficking (e.g., [29]). Finally, the aforementioned research focuses exclusively on deleterious outcomes of violence victimization to the exclusion of examining protective factors, such as (intra)cultural support within the Black community, and positive outcomes, such as posttraumatic growth, including relational, intra-personal, and spiritual growth following violence [8]. Such exclusion makes it difficult to inform strength-based interventions, which may be more culturally congruent for people of Color (e.g., [52]).

### Purpose of the study

Violence victimization is harmful (e.g., [1]), with contextual factors influencing the meaning and trajectory of violence for Black American young adults (e.g., [10]). CBTT (e.g., [19]) offers a scientific framework for empirically examining a wide range of types of victimization and outcomes within the context of inequality. However, the extant literature is limited due to the absence of validated measures of CBTT. Therefore, the purpose of the multi-study report is to develop and validate the Cultural Betrayal Multidimensional Inventory for Black American Young Adults (CBMI-BAYA), with questionnaires including multiple aspects of CBTT: (intra)cultural trust; cultural betrayal; violence victimization—including witnessing and experiencing police violence, physical, sexual, and psychological abuse, sex trafficking and sexual harassment—and racial discrimination; (intra)cultural pressure and (intra)cultural support; and posttraumatic growth.

## Method

### Ethics statement

The Institutional Review Boards (IRBs) of Wayne State University (Study 1 and Study 2) and Virginia State University (Study 1) granted approval for the current research. For Study 1, formal consent was obtained in writing or through indicating consent in the online form prior to beginning the survey, as required by the respective university IRBs. For Study 2, participants indicated consent in the online form prior to beginning the survey.

### Participants: Study 1

Participants (*N* = 342) were Black university students attending either a historically Black/African American university in the Southeastern U.S. or a historically White university in the Midwestern U.S. Participants were average college age (*M* = 20.03 years, *SD* = 3.07), majority women (76.0%; men: 7.9%; other/missing gender: 14.3%), with majority low annual income ($40,000 or less: 92.1%). Inclusion criteria were Black/African American identification and current university enrollment. Participants received course credit and were able to decline to answer any question without penalty. The recruitment period was from 04/10/2018 – 20/09/2020.

The university Institutional Review Boards (IRBs) approved the current study.

### Participants: Study 2

Participants (*N* = 619) were recruited from the Prolific Academic online platform [53,54] and were compensated at a rate of $6.50/hr. Participants were Black/African American young adults (*M* = 24.72 years, *SD* = 3.52 years), with relatively equal gender breakdown between men and women (men: 46.8%; women: 48.0%; non-binary/other: 1.1%). Income ranged from low ($40,000 or lower: 58.1%), mid ($40,000 to $80,000: 21.8%), and high ($80,000 or higher: 15.5%). Inclusion criteria were Black/African American identification, U.S. residence, and age 18 – 30 years old. The recruitment period was from 12/11/2019 – 21/06/2021. The university IRB approved the current study.

### Measures

These data are part of a larger data collection, therefore, only some of the measures are reported here (*PI: Jennifer M. Gómez*).

The Cultural Betrayal Multidimensional Inventory for Black American Young Adults contains 5 questionnaires measuring six constructs. S2 Appendix has the full descriptions of each construct.

#### CBMI-BAYA: (Intra)Cultural Trust Questionnaire.

The pre-validated (Intra)Cultural Trust Questionnaire has 27 items that assess (intra)cultural trust (*see* S3 Appendix). The definition of (intra)cultural trust is the connection—including love, loyalty, responsibility, and solidarity—with other members of the Black community. The instructions ask participants to think about their relationship with the Black community. Items were rated on a Likert scale from 0- not at all to 5- a lot. There were three proposed sub-scales: collective self-esteem {sample item, *I feel connected to the Black community*}, Black identity {sample item: *Being Black is very important to my identity*}, and Black pride {sample item: *I am proud to be a Black person*}, with three additional items assessing strength of (intra)cultural trust over time {sample item: *My identification with the Black community has gotten stronger as I have grown up*}. Continuous mean variables were used in correlational analyses.

#### CBMI-BAYA: Cultural Betrayal Questionnaire.

The pre-validated Cultural Betrayal (CB) Questionnaire has 20 items that assess cultural betrayal (*see* S3 Appendix). Cultural betrayal is defined as violation, harm, or violence perpetrated by a person of the same marginalized group(s). The CB Questionnaire assesses non-violent cultural betrayal. The instructions ask participants to think about things other Black people have done directly to them, that they have witnessed, or that have happened in larger contexts, such as by famous people. The stem for each item is *Throughout your life, how many times has another Black person*…with a Likert scale from 0- none to 4- many times. The three proposed subscales were being accused of acting White or not being Black enough {sample item:...*discredited your viewpoint on issues in the Black community because of other identity(ies) you have (i.e., biracial, female, gay, etc).?*}, back-stabbing behavior/lack of solidarity {sample item:...*exhibited back-stabbing behavior towards you in the workplace (i.e., sabotaged your promotion, etc.)?*}, and anti-Black sentiment {sample item:...*said that racism did not negatively affect the Black community?*}. Continuous mean variables were used in correlational analyses.

#### CBMI-BAYA: Violence & Discrimination Questionnaire.

The pre-validated Violence & Discrimination (V&D) Questionnaire (*see* S3 Appendix) of the CBMI-BAYA assesses: witnessing physical, sexual, and emotional abuse, and police (physical) violence (items 1–4), experiencing physical (item 5), sexual (items 6–7), and emotional abuse (item 14), sexual harassment (item 8), child sexual abuse (item 9), sex trafficking (items 10–11), racial discrimination (items 12–13), and police (physical) violence (item 15). Sample item: *Have you been forced to engage in sexual acts with other people?* Response choices are yes/no. Follow up items assess retrospective time periods (before age 13, between 13 and 17 years old; 18 years old and older), identity of the perpetrator (via a grid: another Black person(s), another person(s) of Color, a White person(s); person(s) close to you, person(s) you were not close to), and identity of recipients of disclosure (same grid as identity of the perpetrator). Therefore, types of abuse and perpetrator (cultural betrayal vs. between-group) can be assessed separately or combined (e.g., experiencing cultural betrayal child abuse: items 5, 6, 7, 8, 9, 10, 11, 14 by another Black person(s)). Lastly, with a response choice of yes/no, item 16 asks *“Have you ever been abused?”*, thus assessing if participants who endorse behavioral items of abuse also label themselves as someone who has been abused. Subscales of the V&D Questionnaire are any victimization {sample item: *Have you seen anyone be made to engage in sexual acts by being physically forced or while they were sleeping or passed out?*}, any abuse {sample item: *Before you were 13 years old, have you had sexual contact (i.e., touching private parts; sexual acts) with a person or people who were at least five years older than you?*}, “ever abused” {sample item: *Have you ever been abused?*}, witnessing any abuse {sample item: *Have you seen anyone be yelled at, called names, or be putdown (i.e., told they’re stupid, worthless, or unlovable)?*}, any physical abuse {sample item: *Have you been slapped, hit, pushed, strangled, or otherwise physically hurt by another person or people?*}, any sexual abuse {sample item: *Have you been made to engage in sexual acts while you were sleeping or passed out?*}, any contact sexual abuse {sample item: *Have you been made to engage in sexual acts by being physically forced?*}, any sexual harassment {sample item: *Have you been spoken to in a sexually provocative manner while walking on the street, at work, or at school (i.e., cat-calling; unwanted flirting)?*}, any sex trafficking {sample item: *Have you been forced to engage in sexual acts with other people?*}, any emotional abuse {sample item: *Have you been yelled at, called names, or been putdown (i.e., told you’re stupid, worthless, or unlovable)?*}, any racial discrimination {sample item: *Have you been called a racial slur (i.e., the N word) in a derogatory manner?*}, any police violence {sample item: *Have you seen police slap, hit, push, strangle, or otherwise physically hurt a person or people in real life (i.e., not on TV or in movies)?*}, and any police violence victimization {sample item: *Have police slapped, hit, push, strangle, or otherwise physically hurt you?*}.

The V&D Questionnaire in Study 1 contains each item immediately followed by the follow up questions, with ‘check all that apply’ response choices. Conversely, Study 2 includes first all items 1–15 (yes/no response choice), followed by the follow up questions for identity of perpetrator and disclosure recipient only for items in which participant responded “yes.” In both studies, item 16 appears after the first 15 items and item follow up questions. The reasons for these changes are as follows: 1) avoid conflating missing data with a “no” response in the follow up items; 2) eliminate unnecessary research participation time in reading and responding to items that are irrelevant to a given participant; 3) reduce post-data collection coding-burden for research team; and 4) reduce participant fatigue and false negative responses by not showing participants follow up items until they have responded yes/no to items 1–15 (*personal communication with Steven J. Ondersma, Fall 2020*). Categorical variables were calculated and used in analyses.

#### CBMI-BAYA: (Intra)Cultural Pressure & (Intra)Cultural Support Questionnaire.

The pre-validated (Intra)Cultural Pressure & Support (ICPS) Questionnaire consists of two measures assessing (intra)cultural pressure (*N* = 21 items) and (intra)cultural support (*N* = 17 items), respectively (*see* S3 Appendix). The total 38 items across both scales were randomized into the single questionnaire, with only participants who endorsed any violence or discrimination on the V&D Questionnaire completing this questionnaire. The instructions ask participants to think about both the Black community generally and Black people specifically when answering the questions. Based upon the Institutional Betrayal Questionnaire [55], the stem for items is *In thinking about the events described in the previous section, did people in the Black community play a role by*.... The Likert scale was from 0- not at all to 4- A lot. The (Intra)Cultural Pressure measure had three proposed subscales: disclosure {sample item:...*telling you that you won’t be believed by school officials, employers, police, and/or therapists because you’re Black?*}, minimization/rejection/retaliation {sample item:...*retaliating against you in some way (i.e., vandalizing your property, beating you up)?*}, and lifetime messages {sample item:...*disapproving of Black people who talked openly about violence in the Black community (i.e., anyone who encouraged no longer supporting singer R. Kelly due to allegations that he sexually abused Black teen girls?)*}. The (Intra)Cultural Support measure also had three proposed subscales: options {sample item:...*helping you think about the pros and cons of telling what happened to formal sources (i.e., school officials, therapists, child protective services)?*}, acceptance/affirmations {sample item:...*saying that even though many people have experienced what you have, it is still not okay?*}, and lifetime messages {sample item:...*supporting social justice movements (for example, #MeToo) that expose the mistreatment of Black people*?}. Continuous mean variables were used in correlational analyses.

#### CBMI-BAYA: Posttraumatic Growth Questionnaire.

The pre-validated Posttraumatic Growth (PTG) Questionnaire had 38 items assessing different intra- and interpersonal strengths (*see* S3 Appendix). When assessed with those who have been victimized, the PTG Questionnaire functions as a measure of posttraumatic growth. The instructions ask participants to think about how on average, they spend their time, identify strengths they have, and utilize ways they cope with hard times. The stem for items is *How much do you*...with a Likert scale between 0- not at all and 4- a lot. There were six proposed subscales: health behaviors/activities {sample item:...*write in a diary or journal?*}, interpersonal connection/reaching out {sample item:...*share your fears with people close to you?*}, showing love/empathy/support {sample item:...*lend emotional support to someone who is struggling?*}, activism {sample item:...*challenge racist, sexist, etc. jokes when you hear them?*}, cognitions/emotions {sample item:...*believe there is good in the world even though there is also bad in the world?*}, and spirituality {sample item:...*visualize a calm place and travel there in your mind (e.g., sitting under a tree by a river)?*}.

#### Brief Betrayal Trauma Survey.

The Brief Betrayal Trauma Survey (BBTS; [56]) is a 12-item questionnaire that assesses witnessing and experiencing physical, sexual, and emotional abuse by close and unclose others. Responses are on a Likert scale from 0- never to 5- more than 100 times. A sample item is: *You were made to have some form of sexual contact, such as touching or penetration, by someone with whom you were very close (such as a parent or lover).* Though a measure of internal consistency is inappropriate given that the BBTS assesses the prevalence of different events, the measure has demonstrated good test re-test reliability in a large, majority White adult community sample [56]. The BBTS has since been used in young adult samples (e.g., [57–59]), samples of Color (e.g., [12,33,45,46]), racially diverse samples (e.g., [48,60]), and a Black/African American young adult sample [43]. Continuous mean variables for the total measure and witnessing and experiencing physical, sexual, and emotional abuse were created and used in analyses.

#### Trauma Symptoms Checklist.

The Trauma Symptoms Checklist is a 40-item questionnaire that assess trauma-related mental and behavioral health (TSC; [61]). Response choices on the Likert scale range from 1- never to 4- often. A sample item is: *How often have you experienced each of the following in the last two months? “Flashbacks” (sudden, vivid, distracting memories).* The TSC was initially validated on a sample of White young women [61] and has since been used in racially diverse samples (e.g., [44,62]). For the current multi-study report, internal consistency was excellent across both studies’ samples, respectively, α = .94, α = .95. A continuous mean variable for the total TSC was created and used in correlational analyses.

#### Composite International Diagnostic Interview: ‘Beliefs and Experiences’ Module.

The Composite International Diagnostic Interview- ‘Beliefs and Experiences’ Module (CIDI; [63]) is a three-item questionnaire that assesses tactile, visual, and auditory hallucinations, with response choices on a Likert scale of 0- never to 4- almost always. A sample item is: *Have you ever had the experience of hearing things other people could not hear, such as noises or a voice?* The CIDI- ‘Beliefs and Experiences’ Module has been used in racially diverse samples (e.g., [60]) and samples of Color [33,49]. Internal consistency ranged from acceptable to good across both studies’ samples, respectively, α = .69, α = .84. A continuous mean variable including all three items was created and used in analyses.

#### Posttraumatic Disorder (PSTD) checklist-5.

The PTSD Checklist-5 [64] consists of 20 items assessing symptoms of PTSD. The response choices were on a Likert scale, with 0- not at all and 5- extremely. A sample item is: *In the past month, how much were you bothered by... being “superalert” or watchful or on guard?* Continuous mean variable was used in correlational analyses. The PTSD Checklist-5 has been used in diverse research samples (e.g., [46,48,62]), and samples of Color (e.g., [45]), including a Black/African American young adult sample [43]. Internal consistency across both studies’ samples, respectively, was excellent, α = .95, α = .95.

### Procedure

#### Scale development.

Multiple steps were involved in scale development. First, the items for the CBMI-BAYA questionnaires were created by the first author based on the relevant literature [4,5,8,9,13,30,55,56,65–78]. Specifically, the first author used Hinkin’s (1998) recommendations for deductive item development. The generated items were directly from unique and combined aspects of literature on trauma, betrayal, and the intersections of racialized identity and culture. This approach is particularly advantageous when coupled with independently well-developed literatures when specific experts on the intersections of the topics do not exist or are limited in number [79]. This resulted in numerous items for the (intra)cultural trust (N = 27), cultural betrayal (N = 20), violence and discrimination (N = 16), (intra)cultural pressure (N = 20)/(intra)cultural support (N = 18), and posttraumatic growth (N = 38) questionnaires. Next, items within each questionnaire, except for the violence and discrimination questionnaire, were randomized using a free online system (https://www.random.org/lists/). In lieu of randomization, the order of the items on the violence and discrimination questionnaire was modeled after the Brief Betrayal Trauma Survey [56].

To gauge content validity, the first author then uploaded the items to Qualtrics software to facilitate content expert review and feedback. Experts were five interdisciplinary race scholars from public health, epidemiology, developmental psychology, trauma psychology, sociology, and women’s studies. General instructions for expert reviewers are found in S1 Appendix. Specific instructions for expert reviewers for each construct included construct definition, examples of the construct, and information for how the construct will be used within the CBMI-BAYA; expert reviewers responded noting for each item if it were unclear, redundant, double-barreled, needs re-wording, should remove, or unimportant, with a final open-ended question soliciting any additional feedback (*for full details, see* S2 Appendix). The first author reviewed the content expert feedback, followed up with experts to gain clarity on feedback when necessary, and revised the pre-validated CBMI-BAYA, delineating proposed subscales for (Intra)Cultural Trust (3 proposed subscales: *collective self-esteem; Black identity; Black pride*; final 3 items proposed to be distinct, as they assess (intra)cultural trust over time), Cultural Betrayal (3 proposed subscales: *acting White accusation; back-stabbing behavior/lack of solidarity; anti-Black sentiment*), Violence & Discrimination (multiple sub-scales, see Measures and Results sections), (Intra)Cultural Pressure (4 proposed subscales: *disclosure; minimization; rejection/retaliation; negative lifetime messages*), (Intra)Cultural Support (4 proposed subscales: *options; acceptance/affirmation; Blackness; positive lifetime messages*), and Posttraumatic Growth (6 proposed subscales: *healthy behaviors/activities; interpersonal connection; showing love/empathy; activism; adaptive cognitions; spirituality*).

#### Research participation.

Following being finalized in the aforementioned process, participants were recruited from three sites: historically White university in the Midwestern U.S., a historically Black university in the Southeastern U.S. (Study 1), and the Prolific Academic online platform for scientifically sound research recruitment (Study 2; [53,54]). Participants recruited from the predominantly White university and Prolific Academic completed the 30-minute online survey at a location of their own choosing. Participants at the historically Black university completed the 30-minute online survey within a lab setting.

### Data analysis plan

#### (Intra)Cultural Trust, Cultural Betrayal, (Intra)Cultural Pressure, (Intra)Cultural Support Questionnaires.

Our analysis approach is an extension of the Procedure. We assessed the factor structure that was developed by the first author and replicated during the content validity review by expert reviewers. Given the *a priori* development of items across scales, their theoretical basis, and results of the content validity process conducted with content experts, we chose to proceed with confirmatory factor analysis (CFA) to assess the factor structure of the CBMI-BAYA (Intra)Cultural Trust, Cultural Betrayal, (Intra)Cultural Pressure, and (Intra)Cultural Support Questionnaires. Following initial assessment of the CFA, item retention was based on two factors: 1) theoretical content and 2) sufficiently large, standardized factor loadings, as compared to the overall pattern of factor loadings for the particular latent model [80]. This approach is advantageous because it considers the fidelity of theoretical boundaries of the construct [81,82], makes no arbitrary assumption about appropriate cutoff scores [80], and considers information beyond the face value of an individual factor loading in item retention decisions [80]. To assess convergent and divergent validity, we additionally ran bivariate, one-tailed correlational analyses with measures that the questionnaires and subscales should be related or unrelated to based on theory—1) (intra)cultural trust and internalized prejudice (divergent validity); 2) cultural betrayal and trauma symptoms (convergent validity); 3) (intra)cultural pressure and internalized racism, trauma symptoms, hallucinations, and PTSD (convergent validity); and 4) (intra)cultural support and internalized racism, trauma symptoms, hallucinations, and PTSD (divergent validity).

#### Violence & Discrimination (V&D) Questionnaire.

For the V&D Questionnaire in Study 1 and Study 2, we ran descriptive statistics for prevalence by item and by subscale for the total sample and by women/men gender. In Study 2, we additionally assessed prevalence of subscales for the follow up items related to identity of the perpetrator(s) and disclosure recipient(s). To statistically assess construct validity for the Violence & Discrimination Questionnaire in Study 1, we ran chi-square analyses and correlational analyses with relevant BBTS [56] items regarding witnessing physical abuse and experiencing physical, sexual, or emotional abuse. To assess convergent validity in both Study 1 and Study 2, we ran one-tailed bivariate correlational analyses between the full measure and subscales and trauma symptoms, hallucinations, and PTSD.

#### Posttraumatic Growth Questionnaire.

The Posttraumatic Growth Questionnaire is a behavioral checklist, as opposed a measure of a latent construct(s) or indicator of different events. To determine construct validity, we utilized our expert reviewers to provide feedback on theoretical coverage and contribution, item clarity, and general appropriateness of items (S2 Appendix). Their feedback, combined with review by the authors, determined the final questionnaire.

## Results

The purpose of the current multi-study report was to create and validate the Cultural Betrayal Multidimensional Inventory for Black American Young Adults (CBMI-BAYA) across two samples. S3 Appendix has the final items by subscale for each questionnaire. To increase ease in interpreting the findings given there are six questionnaires in the CBMI-BAYA, we have reported the results by questionnaire (e.g., (Intra)Cultural Trust Questionnaire: Study 1 results, Study 2 results; Cultural Betrayal Questionnaire: Study 1 results, Study 2 results, etc.

### (Intra)Cultural Trust Questionnaire

#### Study 1.

We examined the hypothesized factor structure by conducting a CFA in R 4.1.0 using the lavaan (v0.6-11) package. We used diagonally weighted least squares (DWLS) estimation, specifying all items as ordered categorical data. DWLS estimation is appropriate when categorical indicators are used to specify latent variables. Specifically, DWLS makes no assumptions about the distributional properties of the indicators, taking into account potential non-normality at the item level [82]. We first evaluated a model that loaded all items to their respective dimensions—collective self-esteem, Black identity, and Black pride. The model fit was acceptable (χ2(249) = 545.70, *CFI* = .99, TLI = .99, RMSEA = .06, *SRMR* = .07). We then reviewed the standardized factor loadings, finding all ranged between.49 to.85. Whereas these values met suggested magnitude (λ ≥ .30) and significance (*p* < .05) [83], we further considered the unique variance associated with each.

Upon reviewing the residual variances, we noted high residual variances (>.70). Recent scale development studies suggest item retention decisions should consider both residual variance and theory (e.g., [81]). Adopting this approach, the second author identified collective self-esteem subscale items 3, 4, 5, 8, 10, 14, and 23 as those having high residual variances. With item 19’s residual variance being.64, this item was also identified. A total of eight items were presented to the first author for review for theoretical contribution of the items, suggestions from content experts, and the contribution of the other items in the subscale. Following her review, these items were removed from the subscale.

We then returned to our CFA approach to assess the factor structure of the remaining 16 items. The model fit was good (χ2(101) = 154.38, *CFI* = .99, TLI = .99, RMSEA = .04, *SRMR* = .05). The standardized factor loadings for the remaining items ranged from.64 to.94, and the residual variance estimates were.11 to.54. Considering these results, we moved forward with the reduced version of the (Intra)Cultural Trust Questionnaire—10 collective self-esteem items, three Black identity items, and three Black pride items (Table 1). One-tailed correlational analyses provided evidence for divergent validity (Table 2).

**Table 1:** (Intra)Cultural Trust Questionnaire CFA Standardized Factor Loadings.

*Collective Self Esteem Subscale*	Study 1	Study 2
11. I like to be in situations where I am surrounded by Black people.	.69	.87
12. Social justice movements, like #BlackLivesMatter, resonate with me as a Black person.	.68	.79
15. I feel a special bond with Black people.	.79	.91
16. I feel like my identity is greater than just myself because I am a member of the Black community.	.74	.85
18. I feel loyal to the Black community.	.86	.94
20. I feel solidarity with the Black community.	.66	.86
21. When I see a Black person in a position of power, I feel happy.	.71	.78
22. I feel hurt when I witness Black people being mistreated by other members of society (i.e., police brutality, mass incarceration).	.73	.76
24. I feel love towards the Black community.	.78	.89
27. I feel connected to other Black people, including individuals I do not know.	.69	.87
***Black Identity Subscale***		
9. Being Black is very important to my identity.	.83	.94
17. I consider myself a member of the Black community.	.88	.90
26. Being Black is very important to me.	.89	.96
***Black Pride Subscale***		
2. I am proud to be a Black person.	.94	.94
6. I have Black Pride.	.89	.96
7. I am happy to be a Black person.	.64	.80

*Note*. CFA = Confirmatory factor analysis.

**Table 2:** (Intra)Cultural Trust Questionnaire: Divergent Validity.

	Collective Self-Esteem *r* *(R*^*2*^)	Black Identity *r* *(R*^*2*^)	Black Pride *r* *(R*^*2*^)
	Study 1, Study 2	Study 1, Study 2	Study 1, Study 2
Internalized Racism	-.19**, -.53*** (.04,.28)	-.21**, -.49*** (.04,.24)	-.18**, -.49*** (.03,.24)

**p < .01.

***p < .001.

#### Study 2.

We used the second study to replicate the findings from the reduced (Intra)Cultural Trust Questionnaire. Following the same analytical approach as Study 1, we used CFA with DWLS estimation to evaluate the factor structure of the subscale. The model fit the data well (χ2(101) = 211.76, *CFI* = .99, TLI = .99, RMSEA = .04, *SRMR* = .04). Standardized factor loadings for this model ranged between.76 and.96, and associated residual variance estimates ranged from.09 to.42 (Table 1). We ran one-tailed correlational analyses to assess divergent validity (Table 2).

### Cultural Betrayal Questionnaire

#### Study 1.

We replicated the procedures outlined above for the (Intra)Cultural Trust Questionnaire, loading the associated items onto the acting White, back-stabbing, and anti-Black sentiment subscales of the Cultural Betrayal Questionnaire. We evaluated a CFA model to assess the factor structure of the subscales. The fit was marginal, (χ2(167) = 1022.33, *CFI* = .95, TLI = .94, RMSEA = .13, *SRMR* = .11). Factor loadings ranged from.54 to.81. Item 11 was the only item with a residual variance estimate above.70. The first author reviewed the item theoretical contribution in comparison to the rest of the items. Both authors agreed to remove this item. Additionally, because of a combination of borderline theoretical contribution and questionnaire relevance, items 2, 5, 10, 11, 12, 13, and 19 were flagged for removal. We removed two items from the acting White subscale, three items from the back-stabbing subscale, and two items from the anti-Black sentiment subscale. Removing these items improved the model fit, (χ2(62) = 160.08, *CFI* = .99, TLI = .98, RMSEA = .07, *SRMR* = .07). Standardized factor loadings ranged from.69 to.82, with residual variance estimates below.70. Given this improvement, we moved forward with the 13-item version of the Cultural Betrayal Questionnaire (Table 3). We assessed convergent validity through one-tailed correlational analyses (Table 4).

**Table 3:** Cultural Betrayal Questionnaire CFA Standardized Factor Loadings.

*Acting White Subscale*	Study 1	Study 2
6. Discredited your viewpoint on issues in the Black community because of other identity(ies) you have (i.e., biracial, female, gay, etc.)?	.72	.71
9. Made fun of you for being successful?	.72	.76
14. Punished you in some way for being yourself?	.72	.79
16. Accused you of betraying the Black community because you spoke out about problems, such as violence within the Black community?	.82	.75
20. Told you that your perspective on an issue affecting the Black community is wrong because it isn’t “Black enough”?	.77	.76
***Back-Stabbing Subscale***		
1. Exhibited back-stabbing behavior towards you in the workplace (i.e., sabotaged your promotion, etc.)?	.71	.78
3. Cheated you (i.e., giving you an unfair grade in school; stealing your money)?	.69	.77
7. Not supported you in a workplace environment?	.82	.79
8. Made you feel unwelcome at Black-organized events (i.e., Black organizations, meet-up groups, societies, etc.)?	.73	.73
***Anti-Black Sentiment Subscale***		
4. Slandered another Black individual on their rise to success in order to get attention?	.72	.80
15. Ridiculed other members of the Black community who are struggling?	.81	.86
17. Spoken negatively about the Black community?	.75	.79
18. Blamed Black people for the oppression they experience (i.e., when rapper, Kanye West, said that slavery was a choice)?	.80	.76

*Note*. CFA = Confirmatory factor analysis.

**Table 4:** Cultural Betrayal Questionnaire: Convergent Validity.

	Acting White *r* *(R*^*2*^)	Backstabbing *r* *(R*^*2*^)	Anti-Black Sentiment *r* *(R*^*2*^)
	Study 1, Study 2	Study 1, Study 2	Study 1, Study 2
Trauma Symptoms	.23**,.38** (.05,.14)	.18**,.30** (.03,.09)	.19**,.26** (.04,.07)

**p < .01

#### Study 2.

The second study confirmed the appropriateness of the item retention decisions in Study 1. The CFA’s model fit was good, (χ2(62) = 175.70, *CFI* = .99, TLI = .99, RMSEA = .06, *SRMR* = .05). Moreover, standardized factor loadings ranged from.71 to.86, and all residual variance estimates were below.70 (Table 3). Evidence for convergent validity was found through one-tailed correlational analyses (Table 4).

### Violence & Discrimination Questionnaire

#### Study 1.

The Violence & Discrimination (V&D) Questionnaire measures different events, as opposed to an underlying construct, therefore, validation steps were different from the (Intra)Cultural Trust and Cultural Betrayal Questionnaires (*see* Data Analysis Plan section). The V&D Questionnaire is scored as categorical variables, with 1- any victimization and 0- no victimization reported. Prevalence rates, assessed for men and women participants only (*N* = 287) ranged from approximately 2% (sex trafficking through exchange of food/shelter) and just over 88% (witnessing physical abuse; Table 5). There were gender differences in prevalence, with Black young women significantly more likely to indicate experiencing sexual harassment and child sexual abuse. At the subscale level (Table 6), over 95% of the total sample reported any victimization (abuse or racial discrimination), with over 85% reporting any abuse and just under 60% reporting any racial discrimination. Gender differences favored Black young women, who had significantly higher rates of any abuse (approximately 85% vs. 70%), any sexual abuse (over 80% vs. under 20%), any contact sexual abuse (almost 1/3^rd^ vs. just over 10%), and any sexual harassment (over 80% vs. just over 10%). Finally, a small proportion of Black young women who experienced any abuse (over 85%) also endorsed having been abused (approximately 27%). Conversely, no young Black men (0%) indicated they had been abused when asked directly, even though over 70% experienced abuse. Importantly, absence of gender differences in other subscales does not indicate lower overall prevalence rates. Without significant gender differences, witnessing any abuse, for instance was astronomically high at over 90% of the sample.

**Table 5:** Study 1 Violence & Discrimination Questionnaire: Item-Level Prevalence Rates.

Items	Total % (N)	Young Women % (N)	Young Men % (N)	χ2 (1)
***Witnessing***				
1. Physical Abuse	88.2% (253)	74.6% (194)	70.4% (19)	.23
2. Sexual Abuse	7.7% (22)	7.3% (19)	7.4% (2)	.00^+^
3. Emotional Abuse	57.1% (164)	56.5% (147)	63.0% (17)	.41
4. Police Violence	28.9 (83)	22.7% (59)	22.2% (6)	.00
***Experiencing***				
5. Physical Abuse	41.1% (118)	41.5% (108)	37.0% (10)	.21
6. Sexual Abuse—Physical Force	19.9% (57)	20.4% (53)	7.4% (2)	2.66
7. Sexual Abuse—Incapacitation	5.9% (17)	6.2% (16)	3.7% (1)	.26^+^
8. Sexual Harassment	73.2% (210)	79.6% (207)	11.1% (3)	58.47***
9. Child Sexual Abuse—Grooming	18.5% (53)	19.29% (50)	3.7% (1)	4.04*
10. Sex Trafficking—Force	11.5% (33)	11.9% (31)	3.7% (1)	1.67^+^
11. Sex Trafficking—Exchange	2.1% (6)	1.2% (3)	3.7% (1)	1.16^+^
12. Racial Discrimination—Verbal	51.2% (147)	45.09% (117)	40.7% (11)	.18
13. Racial Discrimination—Behavioral	35.2% (101)	30.8% (80)	25.9% (7)	.27
14. Emotional Abuse	57.1% (164)	56.5% (147)	63.0% (17)	.41
15. Police Violence	4.2% (12)	1.9% (5)	3.7% (1)	.38^+^
***Endorse ‘Abuse’***				
16. “Ever Abused”	24.7% (71)	27.3% (71)	0.0% (0)	9.80*

Note: Total N includes full sample across genders; due to low N, only binary gender compared

+ At least one cell has count less than 5

*p < .05; ***p < .001

**Table 6:** Study1 Violence & Discrimination Questionnaire: Subscale Prevalence Rates.

Subscales^—^	Item(s)	Total % (N)	Young Women % (N)	Young Men % (N)	χ2 (1)
Any Victimization	1-15	95.4% (274)	96.2% (250)	88.9% (24)	2.99^+^
Any Abuse	5-11, 14-15	85.4% (245)	86.9% (226)	70.4% (19)	5.37*^+^
“Ever Abused”	16	24.7% (71)	27.3% (71)	0.0% (0)	9.80*
Witnessing Any Abuse	1-4	91.0% (260)	91.2% (237)	85.2% (23)	1.02^+^
Any Physical Abuse	5	41.1% (118)	41.5% (108)	37.0% (10)	.21
Any Sexual Abuse	6-11	76.0% (218)	81.9% (213)	18.5% (5)	53.85***
Any Contact Sexual Abuse	6-7, 9-11	30.0% (86)	31.9% (83)	11.1% (3)	5.05*
Any Sexual Harassment	8	73.2% (210)	79.6% (207)	11.1% (3)	58.47***
Any Sex Trafficking	10-11	12.2% (35)	13.1% (34)	3.7% (1)	2.01^+^
Any Emotional Abuse	14	57.1% (164)	56.5% (147)	63.0% (17)	.41
Any Racial Discrimination	12-13	57.1% (164)	56.5% (147)	42.3% (14)	.22
Any Police Violence—Witness, Experience	4, 15	24.7% (84)	22.7% (59)	22.2% (6)	.00
Any Police Victimization	15	4.2% (12)	1.9% (5)	3.7% (1)	.38

Note: Total N includes full sample across genders; due to low N, only binary gender compared

— Measures experiencing victimization, unless otherwise stated

+ At least one cell has count less than 5

*p < .05; ***p < .001

Due to concerns regarding formatting of the Violence & Discrimination Questionnaire noted in the Measures section, follow-up items were not assessed for Study 1. Face validity, and by extension construct validity, was assessed prior to data collection by the first author and expert reviewer feedback (S1 Appendix and S2 Appendix); additionally related to construct validity, chi-square and correlational analyses, respectively, were run for like constructs between the Violence & Discrimination Questionnaire of the CBMI-BAYA and the BBTS [56]. There were no significant differences in reported prevalence of sexual abuse, *χ2* (1) =.01, *p* = .93. Significant differences favored higher prevalence for the CBMI-BAYA com*p*ared to the BBTS [56] for witnessing physical abuse, *χ2* (1) = 14.50, *p* = .000, experiencing physical abuse, *χ2* (1) = 10.09, *p* = .001, and experiencing emotional abuse, *χ2* (1) = 6.81, *p* = .010. Additionally, the full measures were correlated with one another, *r* = .42, *p* < .001, *R*^*2*^ = .18, as were the total selected like items, *r* = .35, *p* < .001, *R*^*2*^ = .12. Finally, further convergent validity for Study 1 was assessed through examining the correlations between the Violence & Discrimination subscales and trauma sym*p*toms, hallucinations, and PTSD. All subscales were correlated with outcome measures (Table 7), thus *p*roviding evidence for convergent validity.

**Table 7:** Study 1 Violence & Discrimination Questionnaire: Convergent Validity.

	Trauma Symptoms *r* *(R*^*2*^)	Hallucinations *r* *(R*^*2*^)	PTSD *r* *(R*^*2*^)
Any Victimization	.38*** (.14)	.24*** (.06)	.20*** (.04)
Any Abuse	.37*** (.14)	.24*** (.06)	.34*** (.12)
“Ever Abused”	.25*** (.06)	.14** (.02)	.27*** (.07)
Witnessing Any Abuse	.25*** (.06)	.15** (.02)	.14** (.02)
Any Physical Abuse	.18** (.03)	.12* (.01)	.21*** (.04)
Any Sexual Abuse	.35*** (.12)	.21*** (.04)	.28*** (.08)
Any Contact Sexual Abuse	.29*** (.08)	.20*** (.04)	.28*** (.08)
Any Sexual Harassment	.27*** (.07)	.12* (.01)	.15** (.02)
Any Sex Trafficking	.21*** (.04)	.16** (.03)	.19*** (.04)
Any Emotional Abuse	.22*** (.05)	.18*** (.03)	.26*** (.07)
Any Racial Discrimination	.18** (.03)	.12* (.01)	.13* (.02)
Any Police Violence— Witness, Experience	.14** (.02)	.11* (.01)	.09* (.01)
Any Police Victimization	.06 (.00)	.16** (.03)	.09* (.01)

*p ≤ .05; **p < .01; ***p < .001

#### Study 2.

Similar to Study 1, the V&D Questionnaire were scored as categorical variables, with 1- any victimization and 0- no victimization reported. Prevalence rates, assessed for men and women participants only (*N* = 261) ranged from approximately 6.1% (witnessing sexual abuse and experiencing sexual abuse while incapacitated) and just over 74% (witnessing physical abuse; Table 8). This range in prevalence is slightly narrower than that of Study 1. In Study 2, there were gender differences in prevalence, with Black young women significantly more likely to experience sexual abuse through physical force, sexual abuse through incapacitation, sexual harassment, and sex trafficking through force. Conversely, Black young men were at significantly higher risk for witnessing and experiencing police violence. At the subscale level (Table 9), over 90% of the total sample reported any victimization (abuse or discrimination), with just under 80% indicating any abuse and just under 64% indicating any racial discrimination. These rates are similar to those found in Study 1. Gender differences in subscales matched those suggested at the item level. Black young women reported significantly more sexual abuse, contact sexual abuse, sexual harassment, and sex trafficking. Notably, almost 1/3^rd^ of women experienced any contact sexual abuse, with over 23% indicating any sex trafficking. Black young men reported significantly more police violence and police victimization, with 40% indicating witnessing or experiencing any police violence. Like in Study 1, absence of gender differences in other subscales similarly does not indicate lower prevalence rates overall. For instance, over 85% of the sample witnessed any abuse.

**Table 8:** Study 2 Violence & Discrimination Questionnaire: Item-Level Prevalence Rates.

Items	Total % (N)	Young Women % (N)	Young Men % (N)	χ2 (1)
***Witnessing***				
1. Physical Abuse	74.2% (196)	69.4% (93)	79.2% (103)	3.33
2. Sexual Abuse	6.1% (16)	6.0% (8)	6.2% (8)	.00
3. Emotional Abuse	79.5% (210)	77.6% (104)	81.5% (106)	.63
4. Police Violence	32.6% (86)	26.9% (36)	38.5% (50)	4.04*
***Experiencing***				
5. Physical Abuse	53.4% (141)	50.7% (68)	56.2% (73)	.78
6. Sexual Abuse—Physical Force	13.6% (36)	20.9% (28)	6.2% (8)	12.18***
7. Sexual Abuse—Incapacitation	6.1% (16)	10.4% (14)	1.5% (2)	9.20*
8. Sexual Harassment	41.3% (109)	61.9% (83)	20.0% (26)	47.88***
9. Child Sexual Abuse—Grooming	16.3% (43)	20.1% (27)	12.3% (16)	2.98
10. Sex Trafficking—Force	12.1% (32)	20.1% (27)	3.8% (5)	16.47***
11. Sex Trafficking—Exchange	6.4% (17)	7.5% (10)	5.4% (7)	.45
12. Racial Discrimination—Verbal	48.5% (128)	53.0% (71)	43.8% (57)	2.04
13. Racial Discrimination—Behavioral	47.7% (126)	47.8% (64)	47.7% (62)	.00
14. Emotional Abuse	64.0% (169)	65.7% (88)	62.3% (81)	.32
15. Police Violence	8.3% (22)	4.5% (6)	12.3% (16)	5.30*
***Endorse ‘Abuse’***				
16. “Ever Abused”	33.7% (89)	38.1% (51)	29.2% (38)	2.30

+ At least one cell has count less than 5

*p < .05; ***p < .001

**Table 9:** Study 2 Violence & Discrimination Questionnaire: Subscale Prevalence Rates.

Subscales^—^	Item(s)	Total % (N)	Young Women % (N)	Young Men % (N)	χ2 (1)
Any Victimization	1-15	90.2% (238)	89.6% (120)	90.8% (118)	.11
Any Abuse	5-11, 14-15	79.2% (209)	80.6% (108)	77.7% (101)	.34
“Ever Abused”	16	33.7% (89)	38.1% (51)	29.2% (38)	2.30
Witnessing Any Abuse	1-4	86.0% (227)	84.3% (113)	87.7% (114)	.62
Any Physical Abuse	5	53.4% (141)	50.7% (68)	56.2% (73)	.78
Any Sexual Abuse	6-11	48.9% (129)	67.2% (90)	30.0% (39)	36.47***
Any Contact Sexual Abuse	6-7, 9-11	25.0% (66)	32.8% (44)	16.9% (22)	8.91**
Any Sexual Harassment	8	41.3% (109)	61.9% (83)	20.0% (26)	47.88***
Any Sex Trafficking	10-11	15.9% (42)	23.9% (32)	7.7% (10)	12.93***
Any Emotional Abuse	14	64.0% (169)	65.7% (88)	62.3% (81)	.32
Any Racial Discrimination	12-13	64.0% (169)	66.4% (89)	61.5% (80)	.68
Any Police Violence—Witness, Experience	4, 15	33.7% (89)	27.6% (37)	40.0% (52)	4.53*
Any Police Victimization	15	8.3% (22)	4.5% (6)	12.3% (16)	5.30*

— Measures experiencing victimization, unless otherwise stated

+ At least one cell has count less than 5

*p < .05; ***p < .001

Study 2 additionally examined item-level prevalence rates by developmental period (Table 10), identity of perpetrator(s) (Table 11), and identity of disclosure recipient(s) (Table 12). These findings indicated that our sample of Black young adults was exposed to violence across the lifespan from a range of cultural betrayal (Black; other POC) and between-group (White) perpetrators. Disclosure rates varied by type of violence/discrimination and identity of perpetrator (Black, other POC, White)—ranging from approximately 13% of those victimized disclosing sexual abuse by force to a White person and over 78% of those exposed disclosing witnessing physical abuse to a Black person. Finally, convergent validity for Study 2 was assessed through correlational analyses between V&D Questionnaire subscales and trauma symptoms, hallucinations, and PTSD. Providing evidence for convergent validity, we found that all subscales were correlated with all outcomes (Table 13).

**Table 10:** Study 2 Violence & Discrimination Questionnaire: Item-Level Prevalence Rates by Developmental Period.

Items	Childhood % (N)	Adolescence % (N)	Young Adulthood % (N)	Before & After March 2020* % (N)
***Witnessing***				
1. Physical Abuse	41.2% (98)	58.4% (139)	53.4% (127)	11.8% (28)
2. Sexual Abuse	2.9% (7)	2.5% (6)	2.5% (6)	0.8% (2)
3. Emotional Abuse	58.4% (139)	68.5% (163)	65.1% (155)	31.9% (76)
4. Police Violence	10.1% (24)	15.1% (36)	27.7% (66)	10.9% (26)
***Experiencing***				
5. Physical Abuse	33.6% (80)	39.5% (94)	29.0% (69)	9.7% (23)
6. Sexual Abuse—Physical Force	9.7% (23)	6.3% (15)	4.6% (11)	1.3% (3)
7. Sexual Abuse—Incapacitation	1.7% (4)	2.5% (6)	4.2% (10)	0.4% (1)
8. Sexual Harassment	14.3% (34)	33.6% (80)	38.7% (92)	17.2% (41)
9. Child Sexual Abuse—Grooming	16.4% (39)	–	–	–
10. Sex Trafficking—Force	8.8% (21)	5.0% (12)	3.8% (9)	0.4% (1)
11. Sex Trafficking—Exchange	0.4% (1)	1.3% (3)	6.3% (15)	1.3% (3)
12. Racial Discrimination—Verbal	23.9% (57)	38.2% (91)	38.7% (92)	2.1% (5)
13. Racial Discrimination—Behavioral	24.8% (59)	34.5% (82)	37.8% (90)	17.6% (42)
14. Emotional Abuse	44.5% (106)	58.0% (138)	48.7% (116)	21.4% (51)
15. Police Violence	0.8% (2)	5.5% (13)	6.3% (15)	1.3% (3)

Note: Childhood = before the age of 13; Adolescence = ages 13 – 17 years; Young Adulthood = ages 18 – 30 years

*Denotes occurrence both before and after the onset of the COVID-19 pandemic, March 2020.

**Table 11:** Study 2 Violence & Discrimination Questionnaire: Item-Level Prevalence Rates for Black, Of Color, White and Close/Unclose Other Perpetrators.

Items	Cultural Betrayal: Black %, % (N, N)	Cultural Betrayal: Other POC %, % (N, N)	Between-Group: White %, % (N, N)
***Witnessing***			
1. Physical Abuse	44.5%, 44.1% (106, 105)	15.1%, 45.4% (36, 108)	17.2%, 53.8% (41, 128)
2. Sexual Abuse	4.2%, 2.5% (10, 6)	1.7%, 3.8% (4, 9)	1.7%, 3.4% (4, 8)
3. Emotional Abuse	50.8%, 50.8% (121, 121)	23.9%, 47.5% (57, 113)	23.9%, 58.0% (57, 138)
4. Police Violence	7.6%, 18.5% (18, 44)	3.4%, 18.9% (8, 45)	6.3%, 28.6% (15, 68)
***Experiencing***			
5. Physical Abuse	41.2%, 20.6% (98, 49)	10.1%, 22.7% (24, 54)	12.2%, 26.9% (29, 64)
6. Sexual Abuse—Physical Force	9.2%, 5.9% (22, 14)	2.5%, 5.5% (6, 13)	2.1%, 6.7% (5, 16)
7. Sexual Abuse—Incapacitation	5.0%, 2.1% (12, 5)	0.8%, 4.2% (2, 10)	2.1%, 2.5% (5, 6)
8. Sexual Harassment	10.1%, 36.6% (24, 87)	7.1%, 28.6% (17, 68)	7.6%, 27.3% (18, 65)
9. Child Sexual Abuse—Grooming	13.9%, 4.2% (33, 10)	2.1%, 7.6% (5, 18)	2.5%, 5.9% (6, 14)
10. Sex Trafficking—Force	8.4%, 5.0% (20, 12)	2.1%, 6.7% (5, 16)	2.1%, 5.5% (5, 13)
11. Sex Trafficking—Exchange	3.4%, 2.9% (8, 7)	2.9%, 3.8% (7, 9)	2.1%, 4.2% (5, 10)
12. Racial Discrimination—Verbal	12.2%, 22.7% (29, 54)	8.8%, 26.1% (21, 62)	12.6%, 43.3% (30, 103)
13. Racial Discrimination—Behavioral	8.0%, 23.9% (19, 57)	8.8%, 29.4% (21, 70)	12.6%, 40.8% (30, 97)
14. Emotional Abuse	49.6%, 25.2% (118, 60)	17.6%, 29.0% (42, 69)	17.6%, 36.19% (42, 86)
15. Police Violence	3.4%, 2.9% (8, 7)	0.4%, 5.5% (1, 13)	3.8%, 5.5% (9, 13)

*Note: Prevalence rates by Close/Unclose Perpetrators are shown in the Table for “Close Other, Unclose Other” (e.g., 5.0%, 10.2% = 5.0% close other perpetrators and 10.2% unclose other perpetrators)

**Table 12:** Study 2 Violence & Discrimination Questionnaire: Item-Level Prevalence Disclosure Rates for Black, Of Color, White and Close/Unclose Other Recipients.

Items	Black Recipient %, % (N, N)	Other POC Recipient %, % (N, N)	White Recipient %, % (N, N)
***Witnessing***			
1. Physical Abuse	78.2%, 19.0% (169, 41)	37.5%, 30.6% (81, 66)	38.4%, 32.4% (83, 70)
2. Sexual Abuse	40.0%, 25.0% (18, 5)	15.0%, 45.0% (3, 9)	20.0%, 35.0% (4, 7)
3. Emotional Abuse	66.4%, 22.3% (152, 51)	33.6%, 31.0% (77, 71)	35.4%, 33.2% (81, 76)
4. Police Violence	44.0%, 39.0% (44, 39)	21.0%, 42.0% (21, 42)	28.0%, 54.0% (28, 54)
***Experiencing***			
5. Physical Abuse	68.2%, 14.6% (107, 23)	35.0%, 25.5% (55, 40)	42.0%, 24.2% (66, 38)
6. Sexual Abuse—Physical Force	60.5%, 39.5% (23, 15)	18.4%, 34.2% (7, 13)	13.2%, 44.7% (5, 17)
7. Sexual Abuse—Incapacitation	45.0%, 35.0% (9, 7)	35.0%, 40.0% (7, 8)	25.0%, 40.0% (5, 8)
8. Sexual Harassment	67.7%, 12.9% (84, 16)	35.5%, 23.4% (44, 29)	33.1%, 23.4% (41, 29)
9. Child Sexual Abuse—Grooming	56.5%, 23.9% (26, 11)	21.7%, 28.3% (10, 13)	15.2%, 39.1% (7, 18)
10. Sex Trafficking—Force	62.2%, 13.5% (23, 5)	29.7%, 29.7% (11, 11)	29.7%, 37.8% (11, 14)
11. Sex Trafficking—Exchange	45.0%, 30.0% (9, 6)	25.0%, 50.0% (5, 10)	30.0%, 50.0% (6, 10)
12. Racial Discrimination—Verbal	68.5%, 15.4% (98, 22)	35.7%, 21.0% (51, 30)	35.0%, 22.4% (50, 32)
13. Racial Discrimination—Behavioral	64.5%, 27.0% (91, 38)	36.9%, 28.4% (52, 40)	34.0%, 29.8% (48, 42)
14. Emotional Abuse	61.3%, 13.4% (114, 25)	31.7%, 24.2% (59, 45)	31.2%, 25.8% (58, 48)
15. Police Violence	68.0%, 24.0% (17, 6)	32.0%, 40.0% (8, 10)	44.0%, 32.0% (11, 8)

*Note: Prevalence rates by Close/Unclose Perpetrators are shown in the Table for “Close Other, Unclose Other” (e.g., 5.0%, 10.2% = 5.0% close other recipients and 10.2% unclose other recipients); prevalence rates taken from those victimized only (e.g., 10% = 10% of those victimized, not 10% of the total sample)

**Table 13:** Study 2 Violence & Discrimination Questionnaire: Convergent Validity.

	Trauma Symptoms *r* *(R*^*2*^)	Hallucinations *r* *(R*^*2*^)	PTSD *r* *(R*^*2*^)
Any Victimization	.43*** (.18)	.27*** (.07)	.38*** (.14)
Any Abuse	.43*** (.18)	.28*** (.08)	.38*** (.14)
“Ever Abused”	.36*** (.13)	.17** (.03)	.34*** (.12)
Witnessing Any Abuse	.19*** (.04)	.15** (.02)	.20*** (.04)
Any Physical Abuse	.31*** (.10)	.20*** (.04)	.29*** (.08)
Any Sexual Abuse	.35*** (.12)	.18** (.03)	.29*** (.08)
Any Contact Sexual Abuse	.30*** (.09)	.13* (.02)	.26*** (.07)
Any Sexual Harassment	.30*** (.09)	.20*** (.04)	.20*** (.04)
Any Sex Trafficking	.25*** (.06)	.12* (.01)	.20*** (.04)
Any Emotional Abuse	.28*** (.08)	.24*** (.06)	.28*** (.08)
Any Racial Discrimination	.37*** (.14)	.19*** (.04)	.28*** (.08)
Any Police Violence— Witness, Experience	.12* (.01)	.15** (.02)	.16** (.03)
Any Police Victimization	.17** (.03)	.23** (.05)	.20** (.04)

*p < .05; **p < .01; ***p < .001

### (Intra)Cultural Pressure Questionnaire

#### Study 1.

Continuing our approach from the (Intra)Cultural Trust and Cultural Betrayal Questionnaires, we first assessed a measurement model with items loaded onto the minimization and disclosure subscales. The CFA’s model fit was good, (χ2(34) = 38.96, *CFI* = .99, *TLI* = .99, *RMSEA* = .03, *SRMR* = .05). Factor loadings ranged from.62 to.90, and most residual variance estimates fell between.19 to.45. Although the residual variance of item 3 was.61, we decided to retain the item because of its sufficient factor loading and theoretical contribution. Moreover, removing this item undermined model fit. Table 14 reflects retention of all items. We found evidence for convergent validity through one-tailed correlational analyses (Table 15).

**Table 14:** (Intra)Cultural Pressure Questionnaire CFA Standardized Factor Loadings.

*Disclosure*	Study 1	Study 2
3. Telling you that you won’t be believed by school officials, employers, police, and/or therapists because you’re Black?	.62	.66
9. Telling you not to tell the police what happened because it will make things worse for the person or people who did this?	.82	.86
21. Telling you not to tell the police what happened because it will make things worse for the Black community as a whole?	.90	.90
22. Communicating messages throughout your life that violence in the Black community should be kept “in-house” and not shared with people outside of the community (i.e., White people)?	.85	.80
33. Telling you not to tell the police what happened because it will make things worse for you?	.88	.86
***Minimize***		
19. Telling you that you should get over what happened?	.78	.76
20. Telling you that you betrayed the Black community because of what you experienced?	.76	.83
23. Telling you what you experienced is not nearly as bad as what some other Black people have experienced?	.74	.81
25. Denying what happened to you (i.e., saying it didn’t happen at all or that it couldn’t have happened the way you’re saying it did)?	.80	.83
26. Blaming you for the trouble that the person or people who did this to you are in?	.87	.88

*Note*. CFA = Confirmatory factor analysis.

**Table 15:** (Intra)Cultural Pressure: Convergent Validity.

	Disclosure *r* *(R*^*2*^)	Minimize *r* *(R*^*2*^)
	Study 1, Study 2	Study 1, Study 2
Internalized Racism	.22**,.26*** (.05,.07)	.18**,.28*** (.03,.08)
Trauma Symptoms	.14*,.30** (.02,.09)	.25**,.37** (.06,.14)
Hallucinations	.21**,.22** (.04,.05)	.27**,.16* (.07,.03)
PTSD	.22**,.33** (.05,.11)	.30**,.44** (.09,.19)

**p < .01.

#### Study 2.

The model fit in this sample was similar to Study 1, (χ2(34) = 109.53, *CFI* = .99, *TLI* = .99, *RMSEA* = .07, *SRMR* = .05). The lower end of the standardized factor loadings were slightly higher—ranging from.66 to.90—and residual variance estimates ranged from.18 to.56. We note that the residual variance for item 3 remained the highest at.56. However, as with Study 1, removing this item undermined model fit. We decided to retain this item and the overall factor structure of the measure (Table 14). One-tailed correlational analyses provided evidence for convergent validity (Table 15).

### (Intra)Cultural Support Questionnaire

#### Study 1.

We assessed a CFA with associated items loading onto the options and acceptance subscales of the questionnaire. The measurement model for this measure revealed excellent model fit, (χ2(19) = 11.30, *CFI* = 1.00, *TLI* = 1.00, *RMSEA* = .00, *SRMR* = .03). For standardized factor loadings, the range fell between.48 to.90. The residual variance estimates were between.18 to.77. Item 6 had the lowest factor loading at.48 and the highest residual variance at.77. Upon consulting with the first author and reviewing information on the theoretical necessity of the item, both authors agreed the item was of value and should be retained for further consideration (Table 16). Moreover, because it was a single item, the concern for context or cuing effects was minimal. As such, this item was retained for Study 2. One-tailed correlational analyses provided some evidence for divergent validity (Table 17).

**Table 16:** (Intra)Cultural Support Questionnaire CFA Standardized Factor Loadings.

*Options*	Study 1	Study 2
13. Talking to you about various ways to get support (i.e., talk to supportive friends and family; tell trusted mentors in community organizations; engage in religion or spirituality; etc.)?	.69	.87
15. Talking through with you the conflict between wanting to protect yourself, the person or people who did this to you, and the Black community from systems that have been known to cause harm (i.e., police)?	.66	.77
***Accept***		
34. Making you feel accepted in the Black community?	.75	.84
35. Telling you what happened was not your fault?	.86	.88
36. Thanking you for trusting them enough to tell them what happened?	.86	.91
37. Telling you that you will be okay?	.90	.88
38. Reminding you that there is so much strength and perseverance within the Black community?	.84	.82

*Note*. CFA = Confirmatory factor analysis.

**Table 17:** (Intra)Cultural Support: Divergent Validity.

	Options *r* *(R*^*2*^)	Accept *r* *(R*^*2*^)
	Study 1, Study 2	Study 1, Study 2
Internalized Racism	.03, -.12* (.00,.01)	-.12*, -.30*** (.01,.09)
Trauma Symptoms	.16*,.18** (.03,.03)	.03,.06 (.00,.00)
Hallucinations	.14*,.16* (.02,.03)	.05,.16* (.00,.03)
PTSD	.18**,.20* (.03,.04)	.09,.04 (.01,.00)

*p < .05.

**p < .01.

***p < .001.

#### Study 2.

The measurement model in this sample revealed excellent fit, (χ2(19) = 17.76, *CFI* = 1.00, *TLI* = 1.00, *RMSEA* = .00, *SRMR* = .02). Standardized factor loadings ranged from.64 to.92. Additionally, all residual variance estimates were below.70, ranging from.16 to.59. As expected, Item 6 had the lowest factor loading at.64 and highest residual variance estimate –.59. We refit the measurement models for Studies 1 and 2 without Item 6. In both cases, the increase in model fit was negligible. Given the excellent model fit overall the samples from both studies and Item 6’s substantive relevance, we retained all items (Table 16). We found some evidence for divergent validity through one-tailed correlational analyses (Table 17).

#### Posttraumatic growth.

The Posttraumatic Growth questionnaire features six subscales: 1) healthy behaviors and activities, 2) interpersonal connection and reaching out, 3) showing love, empathy, and support, 4) activism, 5) cognitions and emotions, and 6) spirituality. Unlike the other CBMI-BAYA questionnaires, this questionnaire is a behavioral checklist rather than a reflective measure. As such, we relied on the enlisted expert reviewers to determine the appropriateness of each behavioral statement. Expert reviewers were empowered to assess items’ conceptual alignment with the literature, identify items that should be removed, present new items for consideration, and make recommendations for increasing the clarity of items (S2 Appendix).

The expert reviewers did not make suggestions for item deletions or additions. However, they did provide critique of items to increase clarity and ensure they adequately captured the intended behavior. For example, the leading stem of item 32 was “Be caring towards people…” Expert reviewers suggested a change to “Show care towards people…” to enhance the behavioral components of the items. The result of this content review process was retention of all 38 Posttraumatic Growth checklist items, following revision, across the aforementioned six subscales (S3 Appendix).

## Discussion

The purpose of the current multi-study report was to develop and validate the Cultural Betrayal Multidimensional Inventory for Black American Young Adults (CBMI-BAYA). Our findings indicate a theoretically and empirically valid multidimensional inventory across questionnaires assessing (intra)cultural trust, cultural betrayal, violence & discrimination, (intra)cultural pressure, (intra)cultural support, and posttraumatic growth. Specifically, the results establish each questionnaire’s status as a unique construct measure, with subscales that provide suitable coverage of the underlying factor structure. Across two studies, we show strong replication of the factor structure for each questionnaire, following the reduction of items using factor analytic methods, correlational analyses for convergent/divergent validity, and/or content expert feedback. Through the lens of cultural betrayal trauma theory (e.g., 19), the CBMI-BAYA provides a scientific, comprehensive, culturally appropriate multidimensional inventory to assesses group dynamics, violence victimization, racial discrimination, and posttraumatic growth behavior for Black American young adults.

### Implications

An ongoing limitation of the CBTT research (e.g., [43,48]) has been the lack of validated, culturally congruent measures of multiple relevant constructs central to CBTT. Thus, the research has been unable to provide the highest level of evidence for or against the theory. As such, the primary implication of the current study is to engender rigorous violence research in diverse Black American young adult populations. Given that all the known empirical research to date has come from Gómez’ research team (*for a systematic review, see* [20]), the CBMI-BAYA additionally provides the necessary tools for independent researchers to test CBTT in Black young adult populations.

The implications of such improved methodological rigor mirror implications of CBTT itself. Specifically, this research ultimately has implications for clinical interventions. Given the long history of oppression in therapy (e.g., [84]), findings from CBMI-BAYA research can inform culturally competent interventions that meet the needs of Black American young adults. Relational cultural therapy [67,71,74,75,85–87], the liberation health framework [88], and other theoretical orientations that incorporate contextual factors (e.g., relationships, sexism, and racism) into case conceptualization and treatment planning, are vital in working with marginalized survivors of violence. Such therapists can use the research from the CBMI-BAYA to assess if and how the larger trends of CBTT, including cultural betrayal and (intra)cultural trust, pressure, and support, impact their individual clients. Moreover, therapists can promote radical healing within and outside of the therapy room by incorporating the five anchors of radical healing [52]: 1) collectivism; 2) critical consciousness; 3) radical hope [89]; 4) strength and resistance; and 5) cultural authenticity and self-knowledge. This is especially important as racism is the ultimate cause of the harm of cultural betrayal in within-group violence in the Black community. Finally, a long-term clinical implication is to use the findings gained from the CBMI-BAYA being tested in multiple diverse Black American Young Adult populations by independent researchers to design community level interventions that address anti-Black racism, other forms of bigotry impacting Black people (e.g., homophobia, Islamaphobia, sexism), cultural betrayal, cultural betrayal trauma, and (intra)cultural pressure, while leveraging strengths related to (intra)cultural trust and support and posttraumatic growth in the Black community [19].

### Limitations & future directions

Future work can build upon the validated CBMI-BAYA. The current study provides support for the unique factor structure of the continuous CBMI-BAYA construct measures and content validity of the Posttraumatic Growth questionnaire. Overall, the results support both the construct validity and nomological validity of the CBMI-BAYA. Though validated across two diverse samples, the CBMI-BAYA should be validated—and adapted as culturally necessary—in other marginalized Black young adult populations, including gender minorities [90,91]. Additionally, the current CBMI-BAYA is lengthy and thus less applicable to all settings (e.g., screening in primary care; [92]. Therefore, a validated short-form of the CBMI-BAYA may be useful. Furthermore, the V&D Questionnaire provides a tremendous amount of information on various forms of violence and discrimination, including developmental period of victimization and race and relational identity of the perpetrator and disclosure recipients. Future work can further utilize this information to answer research questions related to development, revictimization, cumulative trauma, cultural betrayal (e.g., [93]), and high betrayal (e.g., [31]). Additionally, future research can further refine these measures through qualitative inquiry [19] regarding victimization and more specific Likert responses on prevalence (e.g., number of times vs. “a lot”). Finally, with the CBMI-BAYA as a methodological grounding, researchers can create CBMI’s specific to populations of interest (e.g., Desi youth; Chicana/o/x elders; LGBTQ adults of Color; etc.) that retain the tenets of CBTT while being culturally relevant in different populations. In conclusion, the CBMI-BAYA, as a validated series of questionnaires, provides a culturally congruent methodological avenue for independent researchers to test CBTT with Black American young adult populations, thus increasing the scientific rigor of this work.

## Supporting information

S1 Appendix Instructions for Expert Reviewers. (DOCX)

S2 Appendix Questionnaire Construct Explanations for Expert Reviewers. (DOCX)

S3 Appendix Pre-Validated CBMI-BAYA: Final Items Organized by Subscale Within Each Questionnaire. (DOCX)

## References

[1] Adams-Clark AA, Gómez JM, Gobin RL, Noll LK, Delker BC. Impact of interpersonal, family, cultural, and institutional betrayal on adult survivors of abuse. Handbook of Interpersonal Violence and Abuse Across the Lifespan: A project of the National Partnership to End Interpersonal Violence Across the Lifespan (NPEIV). Cham: Springer International Publishing; 2021. p. 4275–301.

[2] Howard RM, Potter SJ, Guedj CE, Moynihan MM. Sexual violence victimization among community college students. J Am Coll Health. 2018;67(7):674–87. doi: 10.1080/07448481.2018.1500474 30257142

[3] Bailey M. Misogynoir in medical media: on Caster Semenya and R. Kelly. Catalyst. 2016;2(2):1–31.

[4] Crenshaw K. Mapping the Margins: Intersectionality, Identity Politics, and Violence against Women of Color. Stanford Law Review. 1991;43(6):1241. doi: 10.2307/1229039

[5] Bent-Goodley TB. Eradicating domestic violence in the African American community: A literature review and action agenda. Trauma Violence Abuse. 2001;2(4):316–30.

[6] Burstow B. Toward a Radical Understanding of Trauma and Trauma Work. Violence Against Women. 2003;9(11):1293–317. doi: 10.1177/1077801203255555

[7] Burstow B. A Critique of Posttraumatic Stress Disorder and the DSM. J Humanist Psychol. 2005;45(4):429–45. doi: 10.1177/0022167805280265

[8] Bryant-Davis T. Thriving in the wake of trauma: A multicultural guide. Bloomsbury Publishing USA; 2005.

[9] Bryant-Davis T, Chung H, Tillman S. From the margins to the center: Ethnic minority women and the mental health effects of sexual assault. Trauma Violence Abuse. 2009;10(4):330–57. 19578029 10.1177/1524838009339755

[10] Gobin RL, Gómez JM. The cultural context of sexual assault and its consequences among ethnic minority women. Handbook of interpersonal violence and abuse across the lifespan: A project of the national partnership to end interpersonal violence across the lifespan (NPEIV). Cham: Springer International Publishing; 2021. p. 3741–64.

[11] Gómez JM, Gobin RL. Black Women and Girls & #MeToo: Rape, Cultural Betrayal, & Healing. Sex Roles. 2019;82(1–2):1–12. doi: 10.1007/s11199-019-01040-0

[12] Klest B, Freyd JJ, Foynes MM. Trauma Exposure and Posttraumatic Symptoms in Hawaii: Gender, Ethnicity, and Social Context. Psychol Trauma. 2013;5(5):409–16. doi: 10.1037/a0029336 24660048 PMC3961142

[13] Tillman S, Bryant-Davis T, Smith K, Marks A. Shattering silence: Exploring barriers to disclosure for African American sexual assault survivors. Trauma Violence Abuse. 2010;11(2):59–70. 20430798 10.1177/1524838010363717

[14] Bryant-Davis T. Cultural considerations of trauma: Physical, mental and social correlates of intimate partner violence exposure. Psychol Trauma. 2010;2(4):263–5. doi: 10.1037/a0022040

[15] Ford JD, Gómez JM. The Relationship of Psychological Trauma and Dissociative and Posttraumatic Stress Disorders to Nonsuicidal Self-Injury and Suicidality: A Review. J Trauma Dissociation. 2015;16(3):232–71. doi: 10.1080/15299732.2015.989563 25758363

[16] Ford JD, Gómez JM. Self-Injury and Suicidality: The Impact of Trauma and Dissociation. J Trauma Dissociation. 2015;16(3):225–31. doi: 10.1080/15299732.2015.989648 25760316

[17] Pole NE, Triffleman EE. Introduction to the special issue on trauma and ethnoracial diversity. Psychol Trauma. 2010;2(1):1.

[18] Gómez JM. Cultural betrayal trauma theory: The impact of culture on the effects of trauma. Blind to Betrayal. 2012. https://web.archive.org/web/20221006011353/https:/sites.google.com/site/betrayalbook/betrayal-research-news/cultural-betrayal

[19] Gómez JM. The cultural betrayal of Black women and girls: A Black feminist approach to healing from sexual abuse. American Psychological Association; 2023.

[20] Giroux DM, Jean-Baptiste KE, Foster J. A systematic review of cultural betrayal trauma theory. Adv J. 2023;4(1).

[21] Hunt J, Eisenberg D. Mental Health Problems and Help-Seeking Behavior Among College Students. J Adolesc Health. 2010;46(1):3–10. doi: 10.1016/j.jadohealth.2009.08.008 20123251

[22] Kessler RC, Amminger GP, Aguilar-Gaxiola S, Alonso J, Lee S, Ustün TB. Age of onset of mental disorders: a review of recent literature. Curr Opin Psychiatry. 2007;20(4):359–64. doi: 10.1097/YCO.0b013e32816ebc8c 17551351 PMC1925038

[23] Buggs SG, Pittman Claytor C, García SJ, Imoagene O, Keith V, Khoshneviss H, et al. Systemic Anti-Black Racism Must Be Dismantled: Statement by the American Sociological Association Section on Racial and Ethnic Minorities. Soc Race Ethnic. 2020;6(3):289–91. doi: 10.1177/2332649220941019

[24] Gómez JM. Microaggressions and the enduring mental health disparity: Black Americans at risk for institutional betrayal. J Black Psychol. 2015;41(2):121–43.

[25] Harrell SP. A multidimensional conceptualization of racism-related stress: implications for the well-being of people of color. Am J Orthopsychiatry. 2000;70(1):42–57. doi: 10.1037/h0087722 10702849

[26] Hicken MT, Kravitz-Wirtz N, Durkee M, Jackson JS. Racial inequalities in health: Framing future research. Soc Sci Med. 2018;199:11–8. doi: 10.1016/j.socscimed.2017.12.027 29325781 PMC5915332

[27] Pascoe EA, Smart Richman L. Perceived discrimination and health: a meta-analytic review. Psychol Bull. 2009;135(4):531–54. doi: 10.1037/a0016059 19586161 PMC2747726

[28] Ray V. A Theory of Racialized Organizations. Am Sociol Rev. 2019;84(1):26–53. doi: 10.1177/0003122418822335

[29] Bureau of Justice Statistics. Characteristics of Suspected Human Trafficking Incidents, 2008-2010. 2011. http://www.bjs.gov/content/pub/pdf/cshti0810.pdf

[30] Hooks b. Teaching to transgress: Education as the practice of freedom. Routledge; 10.1016/j.nedt.2018.03.022 29627758

[31] Freyd JJ. Betrayal trauma: The logic of forgetting childhood abuse. Harvard University Press; 1998.

[32] Freyd JJ. II. Violations of Power, Adaptive Blindness and Betrayal Trauma Theory. Fem Psychol. 1997;7(1):22–32. doi: 10.1177/0959353597071004

[33] Gómez JM. What’s in a Betrayal? Trauma, Dissociation, and Hallucinations Among High-Functioning Ethnic Minority Emerging Adults. J Aggress Maltreat Trauma. 2018;28(10):1181–98. doi: 10.1080/10926771.2018.1494653

[34] Brown AL, Donner JK. The Education of Black Males in a’post-racial’World. New York: Routledge; 2012.

[35] Ferguson AA. Bad boys: Public schools in the making of black masculinity. University of Michigan Press; 2020.

[36] Gilbert KL, Ray R. Why Police Kill Black Males with Impunity: Applying Public Health Critical Race Praxis (PHCRP) to Address the Determinants of Policing Behaviors and “Justifiable” Homicides in the USA. J Urban Health. 2016;93(Suppl 1):122–40. doi: 10.1007/s11524-015-0005-x 26661386 PMC4824696

[37] Dumas MJ. My brother as “problem” neoliberal governmentality and interventions for black young men and boys. Educ Policy. 2016;30(1):94–113.

[38] Van Cleve N. Crook County: Racism and Injustice in America’s Largest Criminal Court. Stanford (CA): Stanford Univ.

[39] Hooks B. Reconstructing black masculinity. Black looks: Race and representation. 1992. p. 87–113.

[40] Jackson II RL. Scripting the black masculine body: Identity, discourse, and racial politics in popular media. State University of New York Press; 2006.

[41] Durkee MI, Gómez JM. Mental health implications of the acting white accusation: The role of cultural betrayal and ethnic-racial identity among Black and Latina/o emerging adults. Am J Orthopsychiatry. 2022;92(1):68–78. doi: 10.1037/ort0000589 34881962 PMC8831462

[42] Gómez JM. Campus sexual harassment, other violence, and racism, oh my! Evidence from Black women undergraduates for a culturally competent university approach to Title IX. Fem Criminol. 2022;17(3):368–83. 36090530 10.1177/15570851211062574 PMC9455890

[43] Gómez JM. Group dynamics as a predictor of dissociation for Black victims of violence: An exploratory study of cultural betrayal trauma theory. Transcult Psychiatry. 2019;56(5):878–94. doi: 10.1177/1363461519847300 31046633

[44] Gómez JM, Freyd JJ. Psychological Outcomes of Within-Group Sexual Violence: Evidence of Cultural Betrayal. J Immigr Minor Health. 2018;20(6):1458–67. doi: 10.1007/s10903-017-0687-0 29288343

[45] Gómez JM. Does ethno-cultural betrayal in trauma affect Asian American/Pacific Islander college students’ mental health outcomes? An exploratory study. J Am Coll Health. 2017;65(6):432–6. doi: 10.1080/07448481.2017.1341896 28617143

[46] Gómez JM. Isn’t It All About Victimization? (Intra)cultural Pressure and Cutural Betrayal Trauma in Ethnic Minority College Women. Violence Against Women. 2019;25(10):1211–25. doi: 10.1177/1077801218811682 30497342

[47] Gómez JM. What’s the harm? Internalized prejudice and intra-racial trauma as cultural betrayal among ethnic minority college students. Am J Orthopsych. 2019;89(2):237–47. 10.1037/ort0000367 30407029

[48] Gómez JM. Cultural Betrayal as a Dimension of Traumatic Harm: Violence and PTSS among Ethnic Minority Emerging Adults. J Child Adolesc Trauma. 2020;14(3):347–56. doi: 10.1007/s40653-020-00314-0 34471453 PMC8357871

[49] Gómez JM. Does Gender Matter? An Exploratory Study of Cultural Betrayal Trauma and Hallucinations in Latino Undergraduates at a Predominantly White University. J Interpers Violence. 2021;36(3–4):NP1375-1390NP. doi: 10.1177/0886260517746942 29295025

[50] Gómez JM. When solidarity hurts: (Intra)cultural trust, cultural betrayal sexual trauma, and PTSD in culturally diverse minoritized youth transitioning to adulthood. Transcult Psychiatry. 2022;59(3):292–301. doi: 10.1177/13634615211062970 34913370 PMC9149028

[51] Richie B. Compelled to crime: The gender entrapment of battered, black women. Routledge; 2018.

[52] French BH, Lewis JA, Mosley DV, Adames HY, Chavez-Dueñas NY, Chen GA, et al. Toward a Psychological Framework of Radical Healing in Communities of Color. Couns Psychol. 2020;48(1):14–46. doi: 10.1177/0011000019843506

[53] Palan S, Schitter C. Prolific.ac—A subject pool for online experiments. J Behav Exp Finance. 2018;17:22–7. doi: 10.1016/j.jbef.2017.12.004

[54] Peer E, Brandimarte L, Samat S, Acquisti A. Beyond the Turk: Alternative platforms for crowdsourcing behavioral research. J Exp Soc Psychol. 2017;70:153–63. doi: 10.1016/j.jesp.2017.01.006

[55] Smith CP, Freyd JJ. Dangerous safe havens: institutional betrayal exacerbates sexual trauma. J Trauma Stress. 2013;26(1):119–24. doi: 10.1002/jts.21778 23417879

[56] Goldberg LR, Freyd JJ. Self-Reports of Potentially Traumatic Experiences in an Adult Community Sample: Gender Differences and Test-Retest Stabilities of the Items in a Brief Betrayal-Trauma Survey. J Trauma Dissociation. 2006;7(3):39–63. doi: 10.1300/j229v07n03_04 16873229

[57] Gómez JM. High Betrayal Adolescent Sexual Abuse and Nonsuicidal Self-Injury: The Role of Depersonalization in Emerging Adults. J Child Sex Abus. 2019;28(3):318–32. doi: 10.1080/10538712.2018.1539425 30403925

[58] Gómez JM, Kaehler LA, Freyd JJ. Are hallucinations related to betrayal trauma exposure? A three-study exploration. Psychol Trauma. 2014;6(6):675–82. doi: 10.1037/a0037084

[59] Gómez JM, Becker-Blease K, Freyd JJ. A Brief Report on Predicting Self-Harm: Is It Gender or Abuse that Matters? J Aggress Maltreat Trauma. 2015;24(2):203–14. doi: 10.1080/10926771.2015.1002651

[60] Gómez JM, Freyd JJ. High Betrayal Child Sexual Abuse and Hallucinations: A Test of an Indirect Effect of Dissociation. J Child Sex Abus. 2017;26(5):507–18. doi: 10.1080/10538712.2017.1310776 28569650

[61] Elliott DM, Briere J. Sexual abuse trauma among professional women: validating the Trauma Symptom Checklist-40 (TSC-40). Child Abuse Negl. 1992;16(3):391–8. doi: 10.1016/0145-2134(92)90048-v 1617473

[62] Gómez JM. Gendered Sexual Violence: Betrayal Trauma, Dissociation, and PTSD in Diverse College Students. J Aggress Maltreat Trauma. 2021;30(5):625–40. doi: 10.1080/10926771.2020.1783737 35527804 PMC9075698

[63] World Health Organization. Composite International Diagnostic Interview (CIDI, Version 1). ‘Beliefs and Experiences Module’. Geneva: World Health; 1990

[64] Blevins CA, Weathers FW, Davis MT, Witte TK, Domino JL. The Posttraumatic Stress Disorder Checklist for DSM-5 (PCL-5): Development and Initial Psychometric Evaluation. J Trauma Stress. 2015;28(6):489–98. doi: 10.1002/jts.22059 26606250

[65] Bonilla-Silva E. Keynote. Paper presented at the 2015 Conference of Ford Fellows. Washington, D.C., 2015.

[66] Bryant-Davis T, Ocampo C. The trauma of racism: Implications for counseling, research, and education. Couns Psychol. 2005;33(4):574–8.

[67] Comstock DL, Hammer TR, Strentzsch J, Cannon K, Parsons J, Ii GS. Relational‐Cultural Theory: A Framework for Bridging Relational, Multicultural, and Social Justice Competencies. J Counsel Dev. 2008;86(3):279–87. doi: 10.1002/j.1556-6678.2008.tb00510.x

[68] Crenshaw K. Demarginalizing the intersection of race and sex: A Black feminist critique of antidiscrimination doctrine, feminist theory and antiracist politics. Univ Chicago Legal Forum. 1989. p. 139–68.

[69] Durkee MI, Williams JL. Accusations of acting white: Links to black students’ racial identity and mental health. J Black Psychol. 2015;41(1):26–48.

[70] Foynes MM, Freyd JJ. The impact of skills training on responses to the disclosure of mistreatment. Psychol Viol. 2011;1(1):66.

[71] Gómez JM, Lewis JK, Noll LK, Smidt AM, Birrell PJ. Shifting the focus: Nonpathologizing approaches to healing from betrayal trauma through an emphasis on relational care. J Trauma Dissociation. 2016;17(2):165–85. doi: 10.1080/15299732.2016.1103104 26460888

[72] Lavariega Monforti J, Sanchez GR. The Politics of Perception: An Investigation of the Presence and Sources of Perceptions of Internal Discrimination Among Latinos. Soc Sci Quart. 2010;91(1):245–65. doi: 10.1111/j.1540-6237.2010.00691.x

[73] Luhtanen R, Crocker J. A Collective Self-Esteem Scale: Self-Evaluation of One’s Social Identity. Pers Soc Psychol Bull. 1992;18(3):302–18. doi: 10.1177/0146167292183006

[74] Miller JB. Toward a new psychology of women. Beacon Press; 2012.

[75] Miller JB, Stiver I. The healing connection: How women form relationships in therapy and in life. Beacon Press; 1997.

[76] Sanchez GR, Espinosa PR. Does the Race of the Discrimination Agent in Latinos’ Discrimination Experiences Influence Latino Group Identity? Soc Race Ethnic. 2016;2(4):531–47. doi: 10.1177/2332649215624237

[77] Sellers RM, Smith MA, Shelton JN, Rowley SAJ, Chavous TM. Multidimensional Model of Racial Identity: A Reconceptualization of African American Racial Identity. Pers Soc Psychol Rev. 1998;2(1):18–39. doi: 10.1207/s15327957pspr0201_2 15647149

[78] Smith CP, Freyd JJ. Institutional betrayal. Am Psychol. 2014;69(6):575–87. doi: 10.1037/a0037564 25197837

[79] Hinkin TR. A Brief Tutorial on the Development of Measures for Use in Survey Questionnaires. Organ Res Methods. 1998;1(1):104–21. doi: 10.1177/109442819800100106

[80] Flora DB, Flake JK. The purpose and practice of exploratory and confirmatory factor analysis in psychological research: Decisions for scale development and validation. Can J Behav Sci. 2017;49(2):78–88. doi: 10.1037/cbs0000069

[81] Heggestad ED, Scheaf DJ, Banks GC, Monroe Hausfeld M, Tonidandel S, Williams EB. Scale Adaptation in Organizational Science Research: A Review and Best-Practice Recommendations. J Manag. 2019;45(6):2596–627. doi: 10.1177/0149206319850280

[82] Nye CD, Drasgow F. Assessing Goodness of Fit: Simple Rules of Thumb Simply Do Not Work. Organ Res Methods. 2010;14(3):548–70. doi: 10.1177/1094428110368562

[83] Hair JF, Black WC, Babin BJ, Anderson RE. Multivariate data analysis. 7th edition. Pearson Prentice Hall; 2009.

[84] Sue DW. Eliminating cultural oppression in counseling: Toward a general theory. J Counsel Psychol. 1978;25(5):419–28. doi: 10.1037/0022-0167.25.5.419

[85] Birrell PJ, Freyd JJ. Betrayal trauma: relational models of harm and healing. J Traum Pract. 2006;5(1):49–63.

[86] Gómez JM. Trainee Perspectives on Relational Cultural Therapy and Cultural Competency in Supervision of Trauma Cases. J Psychother Integr. 2020;30(1):60–6. doi: 10.1037/int0000154 35558686 PMC9094394

[87] Walker M. What’s a Feminist Therapist to Do? Engaging the Relational Paradox in a Post-Feminist Culture. Women Therapy. 2010;34(1–2):38–58. doi: 10.1080/02703149.2011.532689

[88] Martinez DB, Fleck-Henderson A. Social justice in clinical practice: A liberation health framework for social work. Routledge; 2014.

[89] Mosley DV, Neville HA, Chavez‐Dueñas NY, Adames HY, Lewis JA, French BH. Radical hope in revolting times: Proposing a culturally relevant psychological framework. Soc Person Psych. 2019;14(1). doi: 10.1111/spc3.12512

[90] Jauk D. Gender violence revisited: Lessons from violent victimization of transgender identified individuals. Sexualities. 2013;16(7):807–25. doi: 10.1177/1363460713497215

[91] Mizock L, Lewis TK. Trauma in Transgender Populations: Risk, Resilience, and Clinical Care. J Emotion Abuse. 2008;8(3):335–54. doi: 10.1080/10926790802262523

[92] Gómez JM, Partridge RT. But Is It Okay? The Need to Still Ask Black/African American Mothers About Violence Exposure During The COVID-19 Worldwide Pandemic. GJCPP. 2023;14(3). doi: 10.17161/gjcpp.v14i3.22903

[93] Gómez JM. Gender, Campus Sexual Violence, Cultural Betrayal, Institutional Betrayal, and Institutional Support in U.S. Ethnic Minority College Students: A Descriptive Study. Violence Against Women. 2022;28(1):93–106. doi: 10.1177/1077801221998757 33851553 PMC8582003

