# Supplementary material for: Assessing “Friendly Fire”: The development & validation of the Cultural Betrayal Multidimensional Inventory for Black American Young Adults (CBMI-BAYA)
Source: PLOS Ment Health. 2026 Apr 8;3(4):e0000537. doi: 10.1371/journal.pmen.0000537 (PMC13061259; doi:10.1371/journal.pmen.0000537)
Supplement: S1 Appendix — (DOCX) [file pmen.0000537.s001.docx]

**S1 Appendix.** Instructions for Expert Reviewers

**Instructions for Expert Reviewers**

We greatly appreciate you lending your expertise to the development of the Cultural Betrayal Multidimensional Inventory (CBMI) for Black Emerging Adults in the U.S. CBMI (authors: *masked for peer review*). The CBMI is designed to test cultural betrayal trauma theory (CBTT), created by Dr. Gómez.

Please click this link, where you will access the survey, including all instructions: (LINK).

As a reference document, instructions are listed here as well:

Your review should take no longer than 1 hour to complete.

This version of the CBMI consists of 140 items across 6 subscales: (intra)cultural trust, cultural betrayal, cultural betrayal trauma, (intra)cultural pressure, (intra)cultural support, and posttraumatic growth.

You will find information about cultural betrayal trauma theory in the word document that accompanied the invitation email, as well as at http://jmgomez.org .

You will find definitions of each subscale in the word document that accompanied the invitation email, as well as at the top of each subscale that you will review on Qualtrics.

We ask that you input your name, email, and affiliation into the survey for two reasons: 1) so we can follow up with you if we have any questions about your feedback; and 2) you will receive an acknowledgement in publication and/or presentations for your contribution to the development of the CBMI.

Following reading the definition of the construct assessed in the subscale, you will view the subscale. For each item, you will check a box to indicate when an item is unclear, redundant, double-barreled, needs rewording, should be removed, and/or is unimportant.

Following completing this task for all subscales, you will be given an opportunity to write in any thoughts you would like to share about the CBMI (strengths, weaknesses, suggestions, etc.) that were not already covered previously in the review.

You will have access to this Qualtrics survey beginning 31 August 2018. We ask that you complete your review by midnight, Sunday, 9 September 2018.
