# Supplementary material for: Assessing “Friendly Fire”: The development & validation of the Cultural Betrayal Multidimensional Inventory for Black American Young Adults (CBMI-BAYA)
Source: PLOS Ment Health. 2026 Apr 8;3(4):e0000537. doi: 10.1371/journal.pmen.0000537 (PMC13061259; doi:10.1371/journal.pmen.0000537)
Supplement: S2 Appendix — (DOCX) [file pmen.0000537.s002.docx]

**S2 Appendix.** Questionnaire Construct Explanations for Expert Reviewers in Order

**Cultural Betrayal**

- Definition
  - From a fellow minority, violation of (intra)cultural trust in the form of violence, abuse, violation, or other negative occurrences
  - Examples: being rejected by other members of one's minority group; being accused of ['acting White'](https://www.researchgate.net/publication/271727771_Accusations_of_Acting_White_Links_to_Black_Students%27_Racial_Identity_and_Mental_Health) by other racial minorities
- In the CBMI, cultural betrayal items are linked with the stem, How many times has another Black person(s)...and include:
  - rejection by other Black people
  - denial of anti-Black racism
  - anti-Black sentiment
  - within-group discrimination
  - within-group back-stabbing behavior

**(Intra)Cultural Pressure: (I)CP**

**Definition**
Resulting from societal trauma, a negative transformation of (intra)cultural trust; the needs of victims of cultural betrayal trauma are overshadowed by the perceived needs of the perpetrator and/or the entire minority group

**Examples:**
being told by a fellow minority to keep problems "in house" and not disclose to law enforcement, therapists, etc. because doing so would reflect poorly on and/or harm the perpetrator, other members of the minority group, and/or the minority group as a whole (I)CP is different than cultural betrayal in that (I)CP is tied to violence victimization specifically

**In the CBMI,**(I)CP items are linked with violence victimization specifically, with the stem, In thinking about the events described in the previous section, did people in the Black community play a role by . . . and include items related to:  blaming the victim pressure not to disclose in order to protect the perpetrator(s) and/or Black people putting responsibility for victimization and/or the consequences on the victim being unsupportive pressure to keep problems “in-house”

**(Intra)Cultural Trust: (I)CT**

**Definition**
Connection (e.g., dependency, attachment, loyalty, love, and/or responsibility) with other members of one's minority group(s), potentially as a buffer against inequality

**Examples:**the "sweet sense of solidarity" with other minorities, in which there is an expectation of understanding and support; personal connection with the successes, joys, failures, and harms of one's minority group(s)

**In the CBMI,** (I)CT includes items about the above definition, and:connection and solidarity with the Black community expectation of solidarity and understanding. Importance and centrality of Black identity

**Cultural Betrayal Trauma (violence victimization generally)**

- Definition
  - In this context, “trauma” is defined as witnessing or experiencing violence victimization (aka, physical, sexual, psychological abuse). As well as race incident based trauma (aka, racism, racist acts).
  - Violence victimization can take many forms.
- In the CBMI, types of violence victimization are witnessing or experiencing:
  - physical violence
  - police violence
  - sexual abuse (child & adult; via force or incapacitation)
  - sexual harassment
  - (sex) trafficking (being forced to engage in sexual acts with others; trading sexual acts for something, such as shelter)
    - Note: there is a distinction between trafficking and sex work. That distinction is beyond the scope of the CBMI.
  - psychological abuse (being putdown, degraded, etc.)
  - Racist psychological or physical violence

**(Intra)Cultural Support: (I)CS**

·     Definition

o   An extension of (intra)cultural trust. The needs of the victim are as important as the needs of the minority group. Victim is supported in the aftermath of victimization, as well as in the tensions created by inequality (e.g., need to protect the Black community from discriminatory systems)

o   Examples: being told that you are not responsible for protecting the person(s) who victimized you; helping you think of the pros and cons of disclosing to formal sources, including those that have been and/or continue to be discriminatory against Black people

·     In the CBMI, (I)CS items are linked with violence victimization specifically, with the stem, In thinking about the events described in the previous section, did people in the Black community play a role by . . . and include items related to:

o   offering support to the victim

o   reaffirming the victim’s status in the Black community

o   supporting the victim

o   reaffirming that Black solidarity does not have to include acceptance of within-group violence

- supporting movements or individuals that publicly discuss cultural betrayal trauma

**Posttraumatic Growth: PTG**

- Definition
  - Trauma psychology historically has focused on negative outcomes. PTG identifies that wisdom, strength, and positive things (positive mental health, relationships, etc.) can come after violence victimization.
- In the CBMI, PTG items include those related to:
  - self-care
  - relational connection (with self and others)
  - connection to one’s emotions
  - seeking social support/connection
  - doing things one enjoys
  - activism

**Instructions for Each Construct Included:**

Read each item and click the checkbox for:

**unclear:** it is difficult to understand what it means

**redundant:** it is too similar to other items in the subscale

**double-barreled:** it is asking two or more things within a single item

**needs re-wording:** the syntax, grammar, or phrasing is confusing; needs to be re-written

**should remove:** the item is not related to the construct of interest and should be removed

**unimportant:** the item does not seem to be a good measure of the construct

**Final Question:**

Please write in any thoughts you would like to share about the CBMI (strengths, weaknesses, suggestions, etc.) that were not already covered in your review.
