# Supplementary material for: Assessing “Friendly Fire”: The development & validation of the Cultural Betrayal Multidimensional Inventory for Black American Young Adults (CBMI-BAYA)
Source: PLOS Ment Health. 2026 Apr 8;3(4):e0000537. doi: 10.1371/journal.pmen.0000537 (PMC13061259; doi:10.1371/journal.pmen.0000537)
Supplement: S3 Appendix — (DOCX) [file pmen.0000537.s003.docx]

**S3 Appendix.** Pre-Validated CBMI-BAYA: Final Items Organized by Subscale Within Each Questionnaire

**Cultural Betrayal Multidimensional Inventory for Black American Young Adults**

The creators of this questionnaire are two Black psychologists who want to understand Black people’s experiences: both positive and negative. In this questionnaire, you will be asked questions about experiences you have had, as well as how Black people individually and the Black community as a whole has impacted you. There are no right or wrong answers. Please respond as honestly as possible. Your responses will be kept confidential.

**(Intra)Cultural Trust Questionnaire**

| Not at all | Very little | Kind of | Quite a bit | A lot |
| --- | --- | --- | --- | --- |

In this section, we ask you to think about your relationship to the Black community. There are no right or wrong answers to these questions. We are interested in your honest opinions. Your responses will be kept confidential.

Collective Self Esteem Subscale

11. I like to be in situations where I am surrounded by Black people.

12. Social justice movements, like #BlackLivesMatter, resonate with me as a Black person.

15. I feel a special bond with Black people.

16. I feel like my identity is greater than just myself because I am a member of the Black community.

18. I feel loyal to the Black community.

20. I feel solidarity with the Black community.

21. When I see a Black person in a position of power, I feel happy.

22. I feel hurt when I witness Black people being mistreated by other members of society (i.e., police brutality, mass incarceration).

24. I feel love towards the Black community.

27. I feel connected to other Black people, including individuals I do not know.

Discarded

3. I feel connected to the Black community.

4. The ubuntu saying “I am because we are” fits with how I understand myself in the Black community.

5. When a Black person is successful, I feel like it uplifts all Black people.

8. When I am in environments with mostly White people, I feel comfort when I see another Black person there.

10. I am hurt when Black people say negative things about the Black community.

14. I expect other Black people to understand my experiences as a Black person in America (i.e., dealing with racism).

19. When a tragedy happens to members of the Black community (i.e., White person killing Black people in church), it feels like it has happened to me too.

23. I feel that Black people are dependent on each other for our collective success.

Black Identity Subscale

9. Being Black is very important to my identity.

17. I consider myself a member of the Black community.

26. Being Black is very important to me.

Discarded

None

Black Pride Subscale

2. I am proud to be a Black person.

6. I have Black Pride.

7. I am happy to be a Black person.

Discarded Items

None

For Exploratory Analyses: Single Items Assess Stability-Variability of (Intra)Cultural Trust Over Time

1. My identification with the Black community has gotten stronger as I have grown up.

13. My identification with the Black community has gotten weaker as I have gotten older.

25. My identification with the Black community has remained the same my whole life.

Discarded Items

None

**Cultural Betrayal Questionnaire**

In this section, we ask you to think about things that sometimes happen between Black people. These can be: things that happen directly to you, things that you witness or hear about, or things that happen in larger contexts (i.e., by famous people). Please read each question carefully and answer honestly. Your responses will be kept confidential.

*Throughout your life, how many times has another Black person(s)...*

| None | 1 time | A few times | Several times | Many times |
| --- | --- | --- | --- | --- |

Acting White Subscale

6. Discredited your viewpoint on issues in the Black community because of other identity(ies) you have (i.e., biracial, female, gay, etc.)?

9. Made fun of you for being successful?

14. Punished you in some way for being yourself?

16. Accused you of betraying the Black community because you spoke out about problems, such as violence within the Black community?

20. Told you that your perspective on an issue affecting the Black community is wrong because it isn’t “Black enough”?

Discarded Items

11. Said that you act “White”?

13. Accused you of not really being “Black”?

Back-Stabbing Subscale

1. Exhibited back-stabbing behavior towards you in the workplace (i.e., sabotaged your promotion, etc.)?

3. Cheated you (i.e., giving you an unfair grade in school; stealing your money)?

7. Not supported you in a workplace environment?

8. Made you feel unwelcome at Black-organized events (i.e., Black organizations, meet-up groups, societies, etc.)?

Discarded Items

5. Discriminated against you based on other aspects of your identity (i.e., sexism, homophobia, transphobia, etc.)?

10. Been violent towards you?

19. Been unsupportive when you tell them about a racist incident (i.e., laughing at you; denying your experience; telling you that you are overreacting)?

Anti-Black Sentiment Subscale

4. Slandered another Black individual on their rise to success in order to get attention?

15. Ridiculed other members of the Black community who are struggling?

17. Spoken negatively about the Black community?

18. Blamed Black people for the oppression they experience (i.e., when rapper, Kanye West, said that slavery was a choice)?

Discarded Items

2. Said that racism does not negatively affect the Black community?

12. Said racist things against the Black community in Black “safe spaces” (i.e., church; affinity groups)?

**Violence & Discrimination Questionnaire**

In this section, we ask you to think about events that some people experience. Please read each question carefully and answer honestly. Your responses will be kept confidential.

| Yes | No |
| --- | --- |

.

1. Have you seen anyone be slapped, hit, pushed, strangled, or otherwise physically hurt by another person or people?

2. Have you seen anyone be made to engage in sexual acts by being physically forced or while they were sleeping or passed out?

3. Have you seen anyone be yelled at, called names, or be putdown (i.e., told they’re stupid, worthless, or unlovable)?

4. Have you seen police slap, hit, push, strangle, or otherwise physically hurt a person or people in real life (i.e., not on TV or in movies)?

5. Have you been slapped, hit, pushed, strangled, or otherwise physically hurt by another person or people?

6. Have you been made to engage in sexual acts by being physically forced?

7. Have you been made to engage in sexual acts while you were sleeping or passed out?

8. Have you been spoken to in a sexually provocative manner while walking on the street, at work, or at school (i.e., cat-calling; unwanted flirting)?

9. Before you were 13 years old, have you had sexual contact (i.e., touching private parts; sexual acts) with a person or people who were at least five years older than you?

10. Have you been forced to engage in sexual acts with other people?

11. Have you engaged in sexual acts to get something, such as money, food, drugs, or a place to stay?

12. Have you been called a racial slur (i.e., the N word) in a derogatory manner?

13. Have you been the target of mean, inappropriate, or violent behavior because you are Black?

14. Have you been yelled at, called names, or been putdown (i.e., told you’re stupid, worthless, or unlovable)?

15. Have police slapped, hit, push, strangle, or otherwise physically hurt you?

16. Have you ever been abused?

==

For items 1-15 endorsed with “Yes”, participants answer the following before completing item 16:

- 1. How old were you? Yes/No
     1. younger than 13 years old
     2. 13 years old – 17 years old
     3. 18 years old or older
  2. Did this happen before or after March 2020
     1. Before/After
  3. Who did it?

|  | Person(s) Close To You | Person(s) You Were Not Close To |
| --- | --- | --- |
| Black person(s) |  |  |
| Other person(s) of Color |  |  |
| White person(s) |  |  |

d. Have you told anyone what happened? Yes/No

i. If yes:

|  | Person(s) Close To You | Person(s) You Were Not Close To |
| --- | --- | --- |
| Black person(s) |  |  |
| Other person(s) of Color |  |  |
| White person(s) |  |  |

==

Subscales

Any Victimization: 1 – 15

Any Abuse: 5, 6, 7, 8, 9, 10, 11, 15

“Ever Abused”: 16

Witnessing Any Abuse: 1 – 4

Any Physical Abuse: 5

Any Sexual Abuse: 6, 7, 8, 9, 10, 11

Any Contact Sexual Abuse: 6, 7, 9, 10, 11

Any Sexual Harassment: 8

Any Sex Trafficking: 10, 11

Any Emotional Abuse: 14

Any Racial Discrimination: 12, 13

Any Police Violence (witness, experience): 4, 15

Any Police Victimization: 15

Items can also be combined into subscales by cultural betrayal status and developmental period.

Discarded Items

None

**(Intra)Cultural Pressure & Support (ICPS) Questionnaire**

In this section, we ask you to think about both the Black community generally and individual Black people specifically. Please read each question carefully and answer honestly. Your responses will be kept confidential.

*In thinking about the events described in the previous section, did people in the Black community play a role by . . .*

| Not at all | Very little | Kind of | Quite a bit | A lot |
| --- | --- | --- | --- | --- |

**(Intra)Cultural Pressure**

Disclosure

3. Telling you that you won’t be believed by school officials, employers, police, and/or therapists because you’re Black?

9. Telling you not to tell the police what happened because it will make things worse for the person or people who did this?

21. Telling you not to tell the police what happened because it will make things worse for the Black community as a whole?

22. Communicating messages throughout your life that violence in the Black community should be kept “in-house” and not shared with people outside of the community (i.e., White people)?

33. Telling you not to tell the police what happened because it will make things worse for you?

Discarded Items

None

Minimize

19. Telling you that you should get over what happened?

20. Telling you that you betrayed the Black community because of what you experienced?

23. Telling you what you experienced is not nearly as bad as what some other Black people have experienced?

25. Denying what happened to you (i.e., saying it didn’t happen at all or that it couldn’t have happened the way you’re saying it did)?

26. Blaming you for the trouble that the person or people who did this to you are in?

Discarded Items

5. Saying it will be your fault if the person or people who did this got in trouble (i.e., went to prison)?

11. Have you become less comfortable as a member of the Black community since having experiences described in the previous section?

12. Ignoring you when they see you?

16. No longer wanting to hang out with you?

**(Intra)Cultural Support**

Options

13. Talking to you about various ways to get support (i.e., talk to supportive friends and family; tell trusted mentors in community organizations; engage in religion or spirituality; etc.)?

15. Talking through with you the conflict between wanting to protect yourself, the person or people who did this to you, and the Black community from systems that have been known to cause harm (i.e., police)?

Discarded Items

6. Helping you think about the pros and cons of telling what happened to formal sources (i.e., school officials, therapists, child protective services)?

Accept

34. Making you feel accepted in the Black community?

35. Telling you what happened was not your fault?

36. Thanking you for trusting them enough to tell them what happened?

37. Telling you that you will be okay?

38. Reminding you that there is so much strength and perseverance within the Black community?

Discarded Items

1. Saying that even though many people have experienced what you have, it is still not okay?

2. Supporting social justice movements (for example, #MeToo) that expose the mistreatment of Black people?

4. Retaliating against you in some way (i.e., vandalizing your property, beating you up)?

7. Disapproving of Black people who talked openly about violence in the Black community (i.e., anyone who encouraged no longer supporting singer R. Kelly due to allegations that he sexually abused Black teen girls)?

8. Reminding you that what happened is not a reflection of Black people as a whole?

10. Explaining that what happened in the past does not define you as a Black person?

14. Not inviting you to events and social outings anymore?

17. Saying that Black people need to solve problems as a community without the negative influence of outside systems (i.e., child protective services, police, teachers, therapists)?

18. Telling you that abusive and violent behavior was not okay?

24. Providing environments where these kind of experiences do not happen (e.g., in the home)?

27. Communicating that pride and solidarity in the Black community does not mean accepting inappropriate or harmful behavior?

28. Telling you that what you experienced is common?

29. Making fun of Black public figures who have spoken about violence in the Black community (i.e., Anita Hill, Tarana Burke, @RapedAtSpelman, Black women who said Bill Cosby sexually assaulted them, etc.)?

30. Being clear that you are not responsible for protecting the person or people who did this to you?

31. Accepting you?

32. Laughing at you for being upset over what happened?

**Posttraumatic Growth Questionnaire**

In this section, we ask you to think about how you spend your time, strengths you have, and ways you cope with hard times. We are interested in how often you do the following things on average. Please read each question carefully and answer honestly. Your responses will be kept confidential.

| Not at all | Very little | Sometimes | Quite a bit | A lot |
| --- | --- | --- | --- | --- |

*How much do you . . .*

Behavior

1. Write in a diary or journal

2. Write down and/or tell yourself affirmations that uplift you (i.e., sayings like My spirit provides me with all the strength I need).

5. Read a book

8. Write in a journal about past events that have caused you pain (i.e., break up, witnessing or experiencing violence, etc.)

11. Exercise

15. Eat healthy foods

25. Pamper yourself with calming things you like, such as reading a book, watching a movie, drawing, singing, etc.

27. Do things that make you feel safe, calm, free, and/or connected to the world.

Connection

4. Share your fears with people close to you

9. Share a traumatic event with someone you trust?

17. Spend time at community locations when you do not want to be alone (i.e., church, mosque, community center, etc.)

21. Have healthy relationships with other people

22. Reach out to someone who is supportive of you when you are upset

26. Feel emotionally connected with other people

34. Reach out to a group of people (i.e., family, friends) when you are sad

37. Disclose things you have experienced that make you feel ashamed with people close to you

Support

30. Lend emotional support to someone who is struggling

32. Show care towards people who are more vulnerable than you (i.e., children, subordinates at work, etc.)

33. Show love to others through words and/or actions

35. Feel empathy for other people’s pain and suffering

38. Support another person as they try to accomplish their goals

Activism

14. Challenge racist, sexist, etc. jokes when you hear them

18. Initiate serious discussions about violence, discrimination, poverty, or other injustices

31. Engage in activism in your community and/or career (i.e., volunteering, donating goods, attending community events, writing legislators, teaching, etc.)

Cognitions/Emotions

10. Believe there is good in the world even though there is also bad in the world

12. Feel anger, frustration, and/or rage

16. Express anger without hurting yourself or others

19. Believe that any violence, discrimination, or degradation you experienced was not your fault

20. Reflect on how much you have grown in the past week, month, year, or years

23. Think about people you admire to gain strength, security, wisdom, and support (e.g., public artist, political figure, loved one, deceased family member, etc.)

24. Release pain and stress through crying

28. Cry

29. Trust yourself

36. Trust your perception about how a situation happened

Spirituality

3. Pray

6. Visualize a calm place and travel there in your mind (e.g., sitting under a tree by a river)

7. Feel genuinely connected to humanity

13. Meditate, including closing your eyes and taking long, deep breaths

Discarded Items

None
